# *Staphylococcus aureus* Exfoliative Toxin E, Oligomeric State and Flip of P186: Implications for Its Action Mechanism

**DOI:** 10.3390/ijms23179857

**Published:** 2022-08-30

**Authors:** Carolina Gismene, Jorge Enrique Hernández González, Angela Rocio Niño Santisteban, Andrey Fabricio Ziem Nascimento, Lucas dos Santos Cunha, Fábio Rogério de Moraes, Cristiano Luis Pinto de Oliveira, Caio C. Oliveira, Paola Jocelan Scarin Provazzi, Pedro Geraldo Pascutti, Raghuvir Krishnaswamy Arni, Ricardo Barros Mariutti

**Affiliations:** 1 Multiuser Center for Biomolecular Innovation, IBILCE/UNESP, São José do Rio Preto 15054-000, Brazil; 2 Brazilian Synchrotron Light Laboratory (LNLS), Brazilian Center for Research in Energy and Materials (CNPEM), Campinas 13083-970, Brazil; 3 Institute of Chemistry, University of Campinas, Campinas 13083-970, Brazil; 4 Instituto de Física, University of São Paulo, São Paulo 05314-970, Brazil; 5 Laboratory of Genomic Studies, Sao Paulo State University-UNESP, São José do Rio Preto 15054-000, Brazil; 6 Laboratório de Modelagem e Dinâmica Molecular, Instituto de Biofísica Carlos Chagas Filho, Universidade Federal do Rio de Janeiro, Rio de Janeiro 21941-901, Brazil

**Keywords:** staphylococcal exfoliative toxin E, proline flip, homodimerization, X-ray diffraction, molecular dynamics

## Abstract

Staphylococcal exfoliative toxins (ETs) are glutamyl endopeptidases that specifically cleave the Glu381-Gly382 bond in the ectodomains of desmoglein 1 (Dsg1) via complex action mechanisms. To date, four ETs have been identified in different *Staphylococcus aureus* strains and ETE is the most recently characterized. The unusual properties of ETs have been attributed to a unique structural feature, i.e., the 180° flip of the carbonyl oxygen (O) of the nonconserved residue 192/186 (ETA/ETE numbering), not conducive to the oxyanion hole formation. We report the crystal structure of ETE determined at 1.61 Å resolution, in which P186(O) adopts two conformations displaying a 180° rotation. This finding, together with free energy calculations, supports the existence of a dynamic transition between the conformations under the tested conditions. Moreover, enzymatic assays showed no significant differences in the esterolytic efficiency of ETE and ETE/P186G, a mutant predicted to possess a functional oxyanion hole, thus downplaying the influence of the flip on the activity. Finally, we observed the formation of ETE homodimers in solution and the predicted homodimeric structure revealed the participation of a characteristic nonconserved loop in the interface and the partial occlusion of the protein active site, suggesting that monomerization is required for enzymatic activity.

## 1. Introduction

Several strains of *Staphylococci* express exfoliative toxins (ETs), a group of proteins that function as virulence factors and facilitate host invasion [1,2]. These toxins cleave a desmosomal protein, desmoglein 1 (Dsg1), crucial for cell–cell adhesion in the epidermis, thus leading to blistering skin disorders [3,4,5]. Strains of *Staphylococcus aureus*, a major human pathogen, produce at least four ET variants, termed ETA [6,7], ETB [7], ETD [8] and ETE [9,10], the first three being relevant to human health [1]. ETA-producing *S. aureus* strains can cause Staphylococcal scalded skin syndrome (SSSS), a serious skin disease affecting large parts of the body and characterized by early symptoms such as lethargy, malaise, loss of appetite and fever, and followed by erythematous rash and blistering [1]. A more localized form of this affection, termed bullous impetigo, has been linked to strains expressing ETB [1]. Remarkably, it has been established using animal models that skin blistering can be induced by administering sterile filtrates containing the toxins and that the latter are solely responsible for the most pronounced disease manifestations [1,11,12,13]. On the other hand, ETD was identified in *S. aureus* strains collected from furuncles and cutaneous abscesses, and hence is not believed to be related to SSSS or bullous impetigo [1,8]. ETE, the most recently characterized *S. aureus* ET, previously termed ETD-like, was discovered in *S. aureus* isolates from ewe mastitis [9,10]. Interestingly, it was found that ETE degrades Dsg1 expressed in ruminant teat canal epithelia, which suggests that it might provide advantages for mammary tract colonization [14].

ETs are directly responsible for serious diseases affecting livestock animals, which lead to important economic losses. In this sense, it has been found that ET-producing *S. hyicus* strains are associated with exudative epidermitis (EE) outbreaks in pigs [15]. The clinical manifestations include exfoliation, erythema and serous exudation and have been linked to several ETs, e.g., SHETA, SHETB, ExhA, ExhB, ExhC and ExhD [2]. Remarkably, in 2007, an EE outbreak in China was attributed to a highly pathogenic *S. sciuri* strain expressing ExhC [16]. In addition to skin exfoliation in newborn mice, this toxin is able to induce necrosis in multiple cell cultures [17], which suggests that its role in the pathogenesis of infections caused by ExhC-producing *Staphylococci* might not be limited to skin damage.

ETs are classified as glutamyl endopeptidases (GEPs), enzymes of the chymotrypsin-like serine protease (CLSPs) family that cleave peptide bonds following the α-carboxyl group of Glu residues [18]. They bear the CLSP typical catalytic triad (Asp-His-Ser) and their S1 pockets contain two structural elements present in all known GEPs that play a key role in the preference for Glu, i.e., His and Thr/Ser (Thr in ETs) at positions 213 and 190 (chymotrypsin numbering, hereinafter written in italic) [18]. On the other hand, nearly all known ETs possess a distinctive Lys residue at position *216* in lieu of the Ser residue encountered in many GEPs, except for ExhA of *S. hyicus*, which bears Arg at the equivalent position. It has been proposed that the amine group (-NH_3_^+^) of *K216* helps stabilize the carboxyl group of Glu at the P1 position, a role played by the -NH_3_^+^ of the N-terminal residue in other GEPs [18].

**Figure 1:**
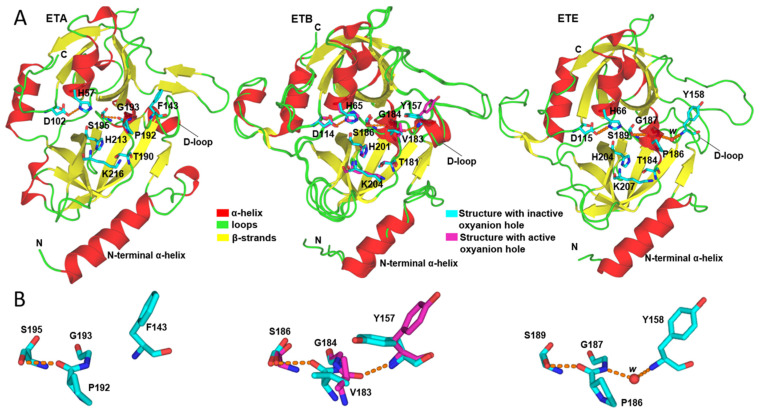
Structural representations of *S. aureus* ETs. (**A**) Cartoon and stick representations of the backbones and key active site residues of ETA (PDB: 1EXF), ETB (PDBs: 1DT2, magenta sticks, and 1QTF, cyan sticks) and ETE (PDB: 5C2Z), respectively. The main structural features of these proteins are depicted, i.e., the D102/114/115-H75/65/66-S195/186/189 catalytic triad, the N-terminal α-helix, the presence in the S1 pocket of residues T190/181/184, H213/201/204 and K216/204/207, which are crucial to the specificity for Glu-P1, atypical flipped conformation of P192/V183/P186(O) atom and the D-loop, which is believed to control the flip. (**B**) Stick representations of the alternate P192/V183/P186(O) conformations observed in the available crystal structures of ETA, ETB and ETE. In all cases, the orange dashed lines represent H-bonds. Residues of ETA are labeled according to the chymotrypsin numbering for ETA, as in PDB: 1EXF. Since the available PDBs of ETB and ETE do not follow the chymotrypsin numbering, residues of these toxins are labeled in sequential order. Numerical labels separated by / correspond to the positions of equivalent residues in ETA, ETB and ETE sequences, in that order. Of note, a water molecule, labeled *w*, forms a water bridge (red sphere) between G187(N) and Y158(N) in the crystal structure of ETE (PDB: 5C2Z). The actual residue numbers in the structure 5C2Z (*n*) took into account 30 residues of the signal peptide and are different from those used in this work (*n-*30).

The available crystal structures of *S. aureus* ETs (PDBs: 1EXF, 1AGJ, 1DUE and 1DUA for ETA; 1DT2 and 1QTF for ETB; and 5C2Z for ETE) have revealed two unique structural features of these proteins: (i) the presence of an N-terminal α-helix containing several charged amino acids, and (ii) an unusual conformation of residues forming the oxyanion hole (Figure 1A,B) [18,19,20,21]. Concerning this last feature, it was first observed in ETA that the *P192-G193* peptide bond is flipped 180° relative to its typical position in other CLSPs [20]. Due to this unusual conformation, which will be termed I (I = inactive), the carbonyl oxygen of *P192* (*P192*(O)) points toward the -OH group of *S195* and forms a hydrogen bond (H-bond) with it (Figure 1B), thereby hindering the access of substrates to the oxyanion hole [20]. Hence, it was proposed that the catalytic activation of ETA would require that *P192*(O) adopts the generally observed conformation, hereinafter termed A (A = active) [19,20]. On the basis of structural analyses, Vath et al. hypothesized that the *P192*(O) flip from conformation I to A could be promoted by an unknown activator that would induce a conformational change in the D-loop (loop_143-153_, Figure 1A) by interacting with the N-terminal α-helix [20]. Likewise, conformation I has been observed for V183(O) and P186(O) of ETB (PDB: 1QTF) and ETE (PDB: 5C2Z), respectively (Figure 1B).

Interestingly, in the structure of ETB (PDB: 1DT2), V183(O) occurs in conformation A (Figure 1B) [21], which suggests that an activator is not essential to promote the flip. It has also been proposed that the equilibrium between conformations A and I of V183(O) may depend on the different crystal forms [21]. Furthermore, the crystal structure of EXI, another ET from *S. pseudintermedius* that hydrolyses canine Dsg1 [22], has been deposited in the Protein Data Bank (PDB: 6E0U) and analysis of the two protein chains reveals that the Pro residue at S1 (P213) also appears in conformation A. These findings support the idea that the oxyanion hole can be properly formed in ETs. However, both the structures (PDBs: 6E0U and 1DT2) have been determined at relatively low resolutions (2.75 and 2.80 Å, respectively), which undermines the previous conclusion.

ETs do not exhibit in vitro proteolytic activity against broad-spectrum substrates of CLSPs, a fact that has been frequently attributed to the unusual conformation of their oxyanion holes [19,20]. Nonetheless, they have been found to display moderate esterolytic and peptidase activities against some small molecules, such as Boc-L-Glu-↓OPh [23] and the peptides α- and β-melanocyte-stimulating hormones (α-MSH and β-MSH), respectively [24]. Therefore, the interaction with Dsg1 is not an absolute requirement for catalytic activity. These lines of evidence, along with the possible existence of alternate conformations of residue *192* observed in the available crystal structures, suggest that the relatively high specificity of ETs, restricted to a few known molecules, may arise from molecular interactions that are independent of the oxyanion hole conformation. 

Our experimental and computational study of ETE provides insights into structural aspects of this toxin that are relevant to the understanding of its action mechanism. Moreover, a high-resolution crystal structure of ETE revealed that P186 exists in two alternate conformations, A and I, thus indicating the occurrence of the P186 flip. Protein–protein docking and molecular dynamics (MD) simulations were combined with small-angle X-ray scattering (SAXS) to predict the conformation of ETE homodimers in solution. The most favorable conformation involves the participation of a nonconserved loop, characteristic of the ETs, and shows that the active site can be partially occluded by the neighboring protein chain.

## 2. Results

### 2.1. Refinement of the ETE Crystal Structure

Recombinant ETE was expressed and purified as described in Materials and Methods. Crystals were obtained with the precipitant sodium formate 4.0 M, pH 6.0 and used in X-ray diffraction experiments. The previously determined ETE structure (PDB: 5C2Z) was crystallized in the space group P 2(1) with cell dimensions a = 49.41, b = 93.14 and c = 50.48 Å and diffracted to 1.96 Å [25]. The current ETE crystal belongs to the space group P 4(3) 2(1) 2 with unit cell dimensions a = 97.53, b = 97.53 and c = 116.35 Å and was refined at 1.61 Å to crystallographic residuals of 0.21/0.24 (Rwork/Rfree) (Table 1).

Coincidentally, both crystal structures of ETE (PDBs: 5C2Z and 8DAX) contain two protein chains in the asymmetric unit (AU); however, the observed protein–protein interactions (PPIs) are different (Figure S1). To assess whether any of the crystallographic homodimers correspond to stable assemblies in solution, the complexes were submitted to the PISA and PRODIGY-CRYSTAL web servers [26,27]. In addition, both putative homodimers were subjected to molecular mechanics generalized Born surface area (MM-GBSA) and umbrella sampling (US) free energy calculations, and the results are summarized in Table S1. As can be observed, none of the homodimers present in the AUs are classified as being stable in solution. According to PRODIGY-CRYSTAL, the PPIs of 5C2Z and 8DAX have a 100 and 65.6% chance, respectively, of being merely the result of crystallographic contacts. Therefore, out of the two structures, the one reported here is more likely to correspond to a stable homodimer, although still classified as crystallographic. Likewise, it ranked better than 5C2Z based on the MM-GBSA effective free energy (Δ*G_eff_*) values determined from 100 ns MD simulations of such systems (Δ*G_eff,100 ns_*). Nonetheless, the free energy estimated from the potential of mean force (PMF) of the new crystal structure, calculated through US, is nearly zero, which underscores that this complex is unstable in solution (Table S1).

### 2.2. Oxyanion Hole and P186 Flip

**Figure 2:**
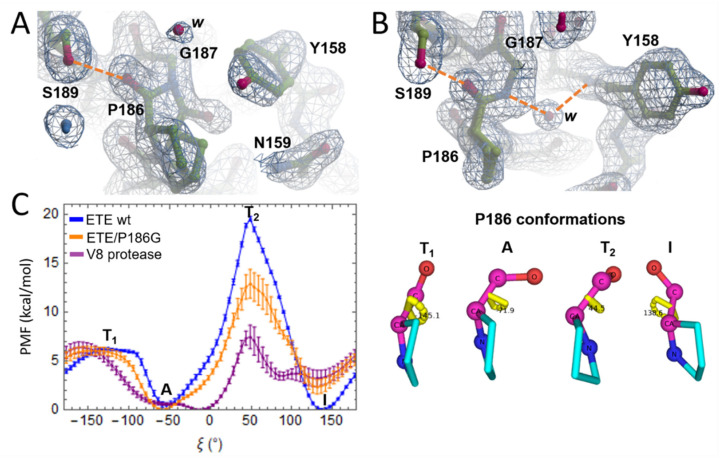
Crystallographic and energetic evidence of P186 dynamic flip in ETE. (**A**) Electron density map of the three-dimensional (3D) structure of ETE (PDB: 8DAX, chain A) showing the occurrence of a 180° flip of P186(O). (**B**) Electron density map of the 3D structure of ETE (PDB: 8DAX, chain B) showing conformation I of P186(O). Residues are labeled and represented as sticks. The electron density is represented as a mesh. H-bonds are depicted as orange dashed lines. Crystallographic water molecules are identified with the letter *w.* (**C**) PMFs for the 360° rotation around the N-CA-C-O dihedrals (ξ) of P186 of ETE wt, G186 of ETE/P186G mutant and G166 of V8 protease (see color legend). The two minima correspond to conformations A and I and the two maxima, T_1_ and T_2_, to the associated energy barriers (see the corresponding P186 conformations on the right panel). Equivalent conformations of G186 and G166 are not shown for brevity’s sake.

The electron density for P186(O) in chain A of the new ETE structure (PDB: 8DAX) shows that this atom can coexist in two alternate conformations A and I (Figure 2A), with occupancies of 0.3 and 0.7, respectively, thus indicating that the latter is relatively more stable. It is worth noting that this crystal structure demonstrates for the first time the occurrence of a dynamic flip between conformations A and I of residue *192* of an ET. Moreover, the respective occupancies are consistent with the fact that conformation I has been more frequently observed in the available crystal structures of ETs than the alternate one. On the other hand, the flip was not detected in the second chain (chain B) of ETE present in the AU (Figure 2A). In this case, P186(O) is pointing toward the oxyanion hole, forming an H-bond with S189(OG) (Figure 2B). Moreover, as in PDB: 5C2Z (Figure 1B), a crystallographic water molecule forms a water bridge between G187(N) and Y158(N) in chain B of PDB: 8DAX. This water molecule is absent when P186 adopts conformation A (compare Figure 2A,B).

It is worth noting that the Y158 side chain occurs in different conformations in each chain of the crystal structure, which seem to be linked to the P186(O) orientation, and will be referred to as conformations 1 (Figure 2A) and 2 (Figure 2B). To assess whether there is an actual correlation in the motion of both residues, we performed five replicate 1 μs MD simulations of ETE in water. Three replicates were started from an ETE structure bearing P186(O) in conformation I and two from a structure with P186 adopting conformation A. In all cases, Y158 was initially oriented as in Figure 2A (conformation 1). First, we observed that almost no transitions between conformations A and I of P186 occurred along the trajectories, thus indicating that crossing the energy barrier separating both states is a rare event when using conventional MD simulations in the microsecond time scale (Figure S2A). Conversely, Y158 sampled different conformations and many transitions during the MD simulations (Figure S2B). Remarkably, the three sampled conformations of Y158 side chain, which include 1 and 2, were observed regardless of the orientation of P186 (Figure S2C,D), which in turn suggests that the motion of these two residues is uncorrelated. This result is further supported by the fact that the Y157 side chain in the crystal structure of ETB (PDB: 1DT2) adopts a conformation different from that of ETE chain B, despite V183(O) and P186(O) being similarly oriented in both structures (compare Figure 1B and Figure 2B).

The inability of conventional MD simulations in the microsecond time scale to properly sample transitions between conformations A and I of P186 indicated the need for enhanced sampling techniques to accurately estimate the free energy associated with that conformational change. Therefore, we conducted US simulations, which allowed us to calculate the PMF corresponding to the 360° rotation of P186 N-CA-C-O dihedral (ξ) and, from this, the relative stability of conformations A and I, as well as the associated energy barriers. Prior to these calculations, we assessed the accuracy of the US free energy protocol by obtaining the PMF for the rotation of the dipeptide L-alanyl-L-proline peptide bond (Figure S3). The predicted free energy associated with the interconversion of *cis* and *trans* conformers is in excellent agreement with the experimental value obtained from the reported equilibrium constant of this process [28]. The major problem with the predictions is the height of the energy barrier, i.e., the free energy of the transition state, which was underestimated by ~6 to 8 kcal/mol. Therefore, when applying the previous protocol in prospective studies, one can expect greater accuracy in the estimates for the free energy difference between conformations corresponding to PMF minima than for the associated transition free energy barriers.

The average PMF for ETE wt shown in Figure 2C (blue PMF profile) displays two energy minima at ξ = −54° and ξ = 136°, corresponding to conformations A and I, respectively, with the latter being slightly more stable than the former by (0.4 ± 0.3) kcal/mol. According to the previous result, these conformations would occur with probabilities of 23 to 45% and 55 to 77%, respectively, in agreement with the two P186(O) occupancies observed in the crystal structure of ETE (Figure 2A). The PMF also displays two energy maxima, T_1_ and T_2_, which define the energy barriers to transition from conformation A to I and vice versa. According to the heights of the energy barriers (~6 and ~19 kcal/mol), the interconversion between both conformations is more likely to occur through T_1_ (energy barrier of 6 kcal/mol), which corresponds to the P186(O) atom pointing in the same direction as the P186 side chain (Figure 2C).

In addition, we calculated the PMF for V8 protease (PDBs: 2O8L), which belongs to a second type of GEP produced by *S. aureus* [18]. This enzyme bears a properly formed oxyanion hole, lacks the N-terminal α-helix and is far more active against the substrate Boc-L-Glu-↓OPh than ETs [19,29]. In agreement with the aforementioned structural features, our calculations show that conformation A of G166(O) (*G192*) is significantly more stable than conformation I (3.2 ± 0.8 kcal/mol) in V8 protease (Figure 2C, purple profile). Interestingly, we also realized, by inspecting previously reported multiple sequence alignments (MSAs), that Gly was always found at position *192* in other *staphylococcal* V8-like GEPs [30,31]. Hence, we generated a model of ETE/P186G mutant through in silico mutagenesis using Pymol [32] and calculated the PMF for the rotation of G186 dihedral (Figure 2C, orange profile). As can be observed, this PMF resembles, to a larger extent, that of V8 protease, with conformation A of G186 being more stable than conformation I by 2.4 ± 0.7 kcal/mol (Figure 2C). This suggests in turn that, if the enzymatic activity of ETE is regulated by the P186 flip, one must expect a significant increase in activity for ETE/P186G. This hypothesis will be evaluated in the next section.

### 2.3. Esterolytic Activity of ETE Wild-Type and ETE/P186G Mutant

**Figure 3:**
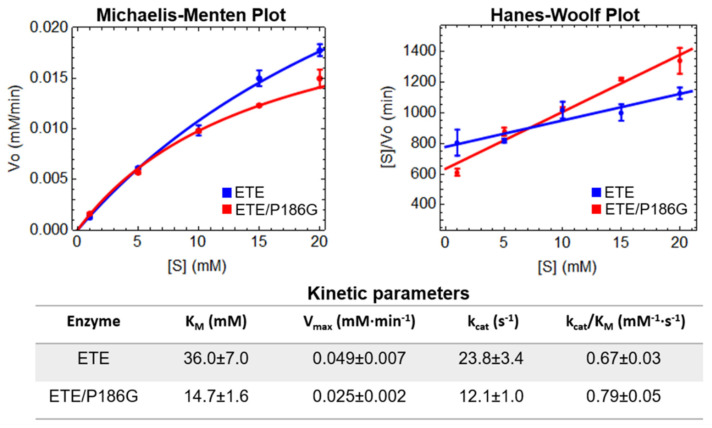
Determination of ETE and ETE/P186G kinetic parameters. The upper panels show the Michaelis–Menten and Hanes–Woolf plots for both enzymes. The table in the lower panel contains the values for the kinetic parameters estimated from the Hanes–Woolf plots through linear regression. V_o_, [S], K_M_, V_max_ and k_cat_ stand for initial velocity, substrate concentration, Michaelis constant, maximal velocity and catalytic constant, respectively. The k_cat_/K_M_ ratio quantifies the catalytic efficiency of each enzyme. Mean values ± standard errors of the mean are shown in all cases.

The esterolytic activity of ETE and ETE/P186G against the synthetic substrate Boc-L-Glu↓-OPh was determined as described in Materials and Methods. In both cases, the initial velocities of the enzymatic reaction at different substrate concentrations follow the characteristic Michaelis–Menten hyperbola (Figure 3). However, due to the relatively high concentrations of Boc-L-Glu-↓OPh used in the assays and the solubility limit of this molecule in 1-4 dioxane, substrate concentrations greater than 20 mM could not be tested and, consequently, enzyme saturation was not reached (Figure 3). In light of these issues, we employed Hanes–Woolf plots [33] to estimate the kinetic parameters of the analyzed enzymes through linear regression (Figure 3).

The estimated kinetic parameters show that the P186G mutation caused a two-fold increase in the association constant of the enzyme and the substrate (1/K_M_) and a two-fold decrease in the maximal velocity (V_max_). Due to these opposing effects, the catalytic efficiencies (k_cat_/K_M_) of ETE and ETE/P186G are very similar, with that of the latter enzyme being slightly higher (Figure 3). Overall, the previous results demonstrate the negligible impact of the P186G mutation on the catalytic activity of ETE, regardless of the expected stabilization of the functional oxyanion hole conformation.

### 2.4. Oligomeric State of ETE in Solution

**Figure 4:**
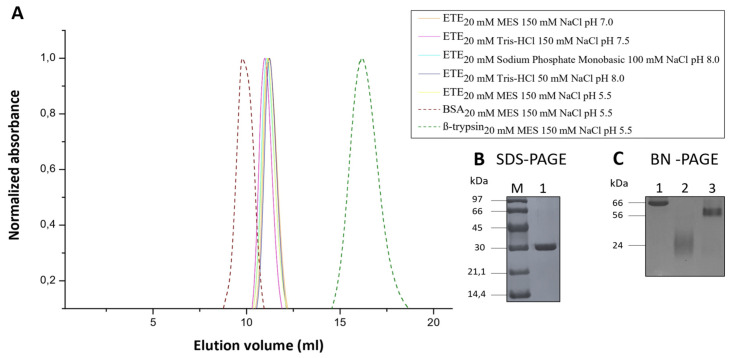
ETE is a dimer in different solution conditions. (**A**) SEC profiles for ETE under different buffering conditions (solid line). BSA and β-trypsin were employed as controls (dash line). Absorbance was measured at 230 nm in all cases, and normalized values are plotted. The overlap of peaks at ~12 mL indicates that ETE elutes in the same oligomeric state in all the tested conditions. (**B**) SDS-PAGE of ETE (1) using the protein marker LMW-SDS Marker KIT—GE Healthcare (M). (**C**) BN-PAGE of ETE (3) using BSA (1) and β-trypsin from bovine pancreas (2) as molecular weight markers. A running buffer containing 25 mM Tris pH 8.3 and 192 mM Glycine was employed for BN-PAGE.

Initial evidence that ETE is a homodimer in solution was obtained based on size-exclusion chromatography (SEC) profiles. Therefore, we decided to further evaluate the oligomeric state of ETE in several solutions comprising different buffers, NaCl concentrations and pH values. As can be observed in Figure 4A, all the chromatograms showed the ETE elution peak at a volume of ~11 mL, thus indicating that the protein occurs in the same oligomeric state in all the tested conditions. Moreover, we obtained the chromatograms for serum albumin (BSA) (MW ~66 kDa) and β-trypsin from bovine pancreas (MW ~24 kDa) and used them as controls to check whether the ETE elution peak corresponds to a dimeric (MW ~60 kDa) or a monomeric protein (MW ~30 kDa). It is evident from the overlap of the different chromatograms that the ETE elution profile is closer to that of BSA (Figure 4A), which confirms that the toxin forms a homodimer in solution.

The previous results were complemented by performing SDS-PAGE and BN-PAGE electrophoresis. As expected, the first method showed that, under denaturing conditions, ETE migrates as a monomer, producing a band at ~30 kDa (Figure 4B). On the other hand, BN-PAGE experiments (Figure 4C) showed the ETE band close to that of BSA monomer, whereas no ETE band corresponding to the molecular weight of β-trypsin was observed. The previous findings are in agreement with the SEC experiments and confirm the formation of ETE homodimers. Similar results are exhibited by ExhC from *S. sciuri*, already described as a homodimer in solution [34], which was used as a control in this study (Text S1 and Figure S4). We also found that ETE/P186G forms homodimers in solution (Figure S5), thus indicating that this mutation does not affect the protein oligomeric state.

**Figure 5:**
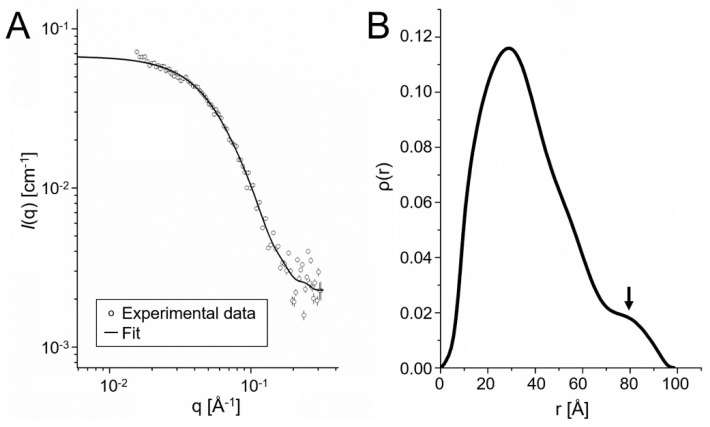
SAXS data for ETE in the buffer solution MES 20 mM, pH 7.0 and 150 mM NaCl. (**A**) Intensity SAXS profile. (**B**) Pair distance distribution function (ρ(r)). *I* and q stand for the intensity and the scattering vector, respectively. The arrow indicates the shoulder in the ρ(r) distribution, which is interpreted as a signature of dimerization.

The oligomeric state of ETE in solution was further studied using SAXS. Figure 5A shows the intensity curve for the toxin in 20 mM MES pH 7.0 and 150 mM NaCl buffer (similar to physiological conditions). Assuming that the protein sample was monodispersed, the indirect Fourier transform (IFT) operation was performed, which provided the distribution of pairs of distances (ρ(r)) (Figure 5B) within the investigated particle [35]. The IFT analysis indicated that ETE has a radius of gyration of (29.8 ± 0.1) Å and a maximum size of ~97 Å. Furthermore, the shape of the ρ(r) distribution obtained is typical for dimeric structures, due to the presence of the shoulder around 80 Å (Figure 5B). These results, in addition to reinforcing the SEC and BN-PAGE data (Figure 4), were also used to support the prediction of the dimeric conformation of ETE in solution.

### 2.5. Prediction of the ETE Homodimer Structure in Solution

As discussed earlier, the contacts formed by the interacting chains in the AUs of the two available crystal structures of ETE were predicted to be merely crystallographic. Therefore, to find the most stable conformation of ETE homodimers in solution we employed the workflow shown in Figure S6, which combined several protein–protein docking algorithms, free energy calculations and SAXS-profile fits (Figure S7). In parallel, we assessed the poor quality of an ETE homodimer model generated by AlphaFold-multimer (Figure S8). The applied computational approaches ultimately led to the identification of a single pose (hereinafter referred to as ETE_2_-CP-0, where CP-0 stands for ClusPro [36] pose 0) as the most stable conformation of ETE homodimers in solution (Table S2). Moreover, the proposed conformation passed subsequent tests aiming to assess the stability of the complex on the basis of its predicted PPI (Table S3). Furthermore, SPPIDER [37] predicted interface-forming residues (IFRs) at the complex interface (Table S3). Altogether, these results strongly suggest that ETE_2_-CP-0 is the most likely conformation of ETE homodimers in solution.

**Figure 6:**
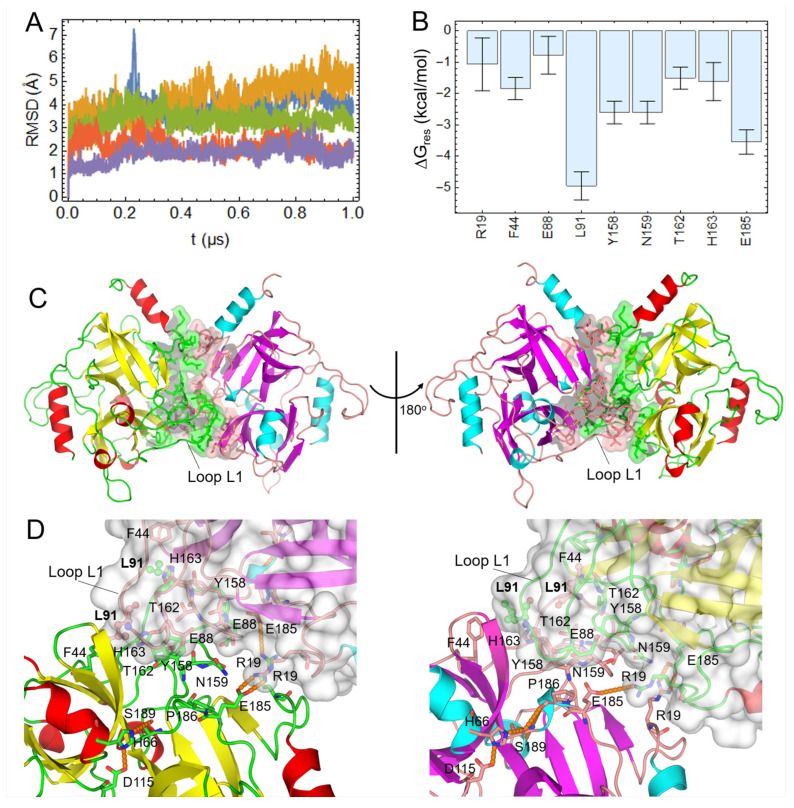
MD-based and structural analyses of the predicted structure of ETE homodimers (ETE_2_-CP-0). (**A**) Backbone root mean squared deviation (RMSD) profiles for the five replicate MD simulations of the ETE homodimer. The initial structure (*t* = 0) was taken as reference for the RMSD calculations. (**B**) Per-residue free energies for the main hotspots of the homodimer interface calculated using MM-GBSA. (**C**) Two views of ETE homodimer upon 180° rotation around the vertical axis. The protein structure is represented as cartoons, with secondary structure elements being colored differently, i.e., red/cyan for α-helices, yellow/magenta for β-strands and green/salmon for loops in chains A/B. Interface residues are shown in surface and stick representations. (**D**) Two views of the complex interface displaying interface hotspot and active site residues as sticks. The main hotspot L91 of the homodimer interface is highlighted in sticks and spheres. H-bonds are represented as orange dashed lines.

The ETE_2_-CP-0 conformation remained relatively stable along five replicate 1 μs MD simulations, as can be inferred from the backbone RMSD profiles shown in Figure 6A. Moreover, the mean Δ*G_eff_* value for the homodimer, calculated by averaging the results from the replicate MD simulations, is −57 ± 2 kcal/mol. This value is consistent with that initially estimated from a single 1 μs MD simulation (−56.2 kcal/mol, Table S2), which reinforces the stability of the homodimer during all the independent MD simulations. Hotspot residues at homodimer PPI, i.e., those largely contributing to the binding process, were also identified by performing MM-GBSA per-residue free energy decomposition (Figure 6B). Two of these residues, R19 and E185, can form intermolecular H-bonds and salt bridges, which explains their favorable energy contribution. On the other hand, L91, Y158 and N159 possess large van der Waals free energy contribution, thus indicating the importance of hydrophobic interactions at the predicted PPI. Interestingly, L91 was identified as the main hotspot of ETE_2_-CP-0, in agreement with previous results pointing out that it is an IFR (Figure 6B and Table S3).

Figure 6C shows a structural representation of the homodimer conformation ETE_2_-CP-0. Like the vast majority of protein homodimers in nature, the proposed structure displays C_2_ symmetry [38], i.e., it is symmetric upon 180° rotation with respect to the indicated vertical axis (Figure 6C). It is worth noting that the proposed model significantly diverges from the crystallographic homodimers present in the AU of PDB structures: 8DAX and 5C2Z, as confirmed by the different composition of interface residues and heavy atom RMSD values (Table S4). Furthermore, the predicted PPI involves residues like Y158 and E185, which are close to P186 and were identified as hotspots of the homodimer interface (Figure 6B,D). An interesting structural feature of the ETE_2_-CP-0 structure is the involvement in the PPI formation of a protruding loop, termed L1 (Figure 6C,D). Of note, this loop contains L91, the main interface hotspot residue (Figure 6B,D). To study the conservation of loop L1 among CLSPs, a structural alignment of different ETs and other proteins of the S1 family was carried out (Figures S9 and S10). It became clear that loop L1 corresponds to an insertion of several amino acids that were only observed in ETs. The equivalent regions in the other CLSPs form significantly shorter loops. A second insertion corresponding to another loop, termed L2, was also found in ETE, ETB and EXI, but it is not part of the predicted homodimer PPI. These results suggest that loop L1 might be a distinctive structural feature of ETs that allows the formation of homodimers.

Finally, we used template-based modeling, in silico mutagenesis and MD simulations to predict the structure of monomeric ETE in complex with a peptide matching the sequence of Dsg1 EC3-EC4 linker (Figure S11). Then, by superimposing this complex onto ETE_2_-CP-0, we realized that loop L1 encroaches into the Sn’ side of the neighboring chain’s active site, thus sterically hindering the full accommodation of the substrate’s peptide. Therefore, it is likely that ETE must undergo monomerization in order to become activated. 

## 3. Discussion

ETs possess several unique features among CLSPs, i.e., a relatively high specificity for Dsg1, a densely charged N-terminal α-helix and a nonfunctional oxyanion hole due to a flipped conformation of residue *192* in most of the available crystal structures [18,19,20,21]. To date, the activation mechanism of these toxins remains largely unknown. Moreover, the direct role of the unusual conformation of residue *192* in the activation process cannot be inferred based on the controversial findings reported in the literature, e.g., the existence of crystal structures of ETs with functional oxyanion holes (PDBs: 1DT2 and 6E0U) [21]. Here, we provide evidence that sheds light on some of these issues by establishing connections between the oligomeric state and the oxyanion hole conformations with the action mechanism of ETE [14,25].

We found by inspecting a newly reported crystal structure of ETE (PDB: 8DAX) that the P186 residue of chain A occurs in two alternate conformations, I and A, corresponding to P186(O) oriented toward the catalytic Ser or rotated 180°. Furthermore, crystallographic evidence that P186(O) can coexist in alternate positions was supported by free energy calculations, which demonstrated that conformation I is marginally more stable than conformation A (~0.4 kcal/mol) and that the associated energy barrier separating them is only 6 kcal/mol high. For comparison purposes, we calculated the free energy barrier associated with the *cis-trans* isomerization of the peptide bond of L-alanyl-L-proline in bulk water [28], which turned out to be two times higher. Overall, our results show that the P186 flip is a dynamic event that can occur, in principle, without being promoted by an effector molecule and hence is unlikely to be the main structural feature controlling the ETE enzymatic activity.

More evidence in this regard was obtained from the analysis of the ETE/P186G mutant. Based on the presence of Gly at position *192* of staphylococcal V8-like GEPs [30,31], which possess functional oxyanion holes and higher esterolytic activities than ETs [18,29], we assessed whether this ETE variant would acquire V8-like properties. The free energy calculations performed in our work confirmed that the P186G mutation significantly stabilizes conformation A (2.4 ± 0.7 kcal/mol more stable than conformation I). Moreover, the esterolytic assays showed that it causes a two-fold increase in the association constant of the enzyme and the substrate. This modest affinity increase is likely the result of having the entire population of ETE/P186G with a preformed oxyanion hole. Hence, there is no energetic penalty associated with shifting the equilibrium from conformation I to A during substrate binding, as occurs for the wild-type enzyme. However, it was also found that the catalytic constant of the mutant is almost half that of ETE. Consequently, the catalytic efficiencies against Boc-L-Glu-↓OPh of ETE and ETE/P186G are approximately equal, thus reinforcing the slight impact of residue 186 flip on the enzymatic activity.

Furthermore, it was determined that ETE and ETE/P186G form homodimers in aqueous solution, a conclusion that was derived by using different experimental approaches, i.e., SEC, BN-PAGE and SAXS. This was not a surprising finding per se, since ExhC, an ET from *S. sciuri*, had already been reported as a homodimer in solution using some of the previous approaches [34]. Nonetheless, the fact that at least two different toxins form homodimers suggests that this might be a general property of ETs, probably playing some functional role.

Interestingly, although the two available crystal structures of ETE possess two contacting protein chains in the AU, several computational analyses performed here revealed that none of them corresponds to a stable biological assembly. Therefore, we combined different computational approaches to predict the most favorable conformation of ETE homodimers. Of note, the proposed structure displays partially occluded active sites at the Sn’ region, a feature that suggests that homodimers may show reduced or no catalytic activity and that monomerization would be a necessary step for ETE to become fully activated. This last event could be favored, for example, by interacting with Dsg1. The esterolytic of ETE against Boc-Glu↓-OPh poses additional questions on the ability of homodimers to directly cleave small molecules that do not bind extensively to the occluded Sn’ side of the active site. Furthermore, the crystallization of ETE in assemblies different from the one found to be stable in solution and the relatively low affinity estimated by PISA on the basis of the predicted complex structure (Δ*G_bind_* = −3.1 kcal/mol) indicate the formation of a labile homodimer that could readily dissociate under certain conditions. This evidence reinforces that monomerization is a plausible step that could initiate the activation of this enzyme.

The partial occlusion of the ETE active site in the predicted homodimer is caused by loop L1. Moreover, the main hotspot of the PPI, L91, lies in the aforementioned loop, thus suggesting that the latter is essential for the homodimer formation. This hypothesis can be explored using site-directed mutagenesis and deletion of loop residues, which will help assess the influence of the loop on the dimerization of ETE. Of note, our results show that the protruding loop L1 seems to be a characteristic structural element of several ETs, not found in other CLSPs with available crystal structures. Therefore, it is likely to play a key role in the homodimerization of other ETs. Future studies must be carried out to evaluate the oligomeric state of all known ETs and the involvement of their loops L1 in the formation of the PPIs.

Hanakawa et al. proposed the existence of an exosite in the ETs responsible for their specific interaction with Dsg1 EC3 and subsequent activation [4]. Our results pose new questions about the interplay between homodimerization of ETs and the recognition of EC3 via exosites and whether the latter overlap, to some extent, with the homodimer interface. Should that be the case, monomerization would be necessary not only to expose the active site but to enable the interaction of ETs with their natural substrate. Further experiments are required to establish the oligomeric state of catalytically inactive ETs (e.g., ETE/S189A) in the presence of Dsg1 and to locate their exosite.

## 4. Materials and Methods

### 4.1. Protein Expression and Purification

*Escherichia coli* BL21(DE3)-T1R competent cells were transformed with pD441-ETE and pET28a(+)-ETE/P186G (GenScript) expression vectors. The selected bacterial colonies were grown overnight at 37 °C in Lysogeny Broth (LB) medium supplemented with kanamycin (50 mg/mL). Subsequently, the cultures were diluted 100-fold with a fresh LB medium containing kanamycin (50 mg/mL) and incubated at 37 °C. When the optical density measured at 600 nm (OD_600_) reached 0.5, expression was induced with 0.4 mM IPTG for 5 h at 30 °C. The cells were collected by centrifugation at 2600× *g* for 10 min at 4 °C and homogenized by sonication in lysis buffer containing 5 mM NaHPO_4_, pH 7.7, 400 mM NaCl, 10 mM imidazole and 10% (*v*/*v*) glycerol. The lysed cells were centrifuged at 15,000× *g* for 30 min at 4 °C, and both supernatants with a large amount of ETE and ETE/P186G were subjected to affinity chromatography using a Ni2+-Sepharose column (Bio-Rad), according to the manufacturer’s instructions. The protein concentration was determined spectrophotometrically at approximately 10 mg/mL (Biomate 3S—Thermo scientific spectrophotometer), and additional purification steps were performed for ETE and ETE/P186G in 20 mM MES pH 7.0 and 150 mM NaCl buffer (and other buffering conditions for ETE) on an AKTA system purifier by SEC using a Superdex G75 10/300 GL column (GE—Healthcare Life Sciences) at a flow rate of 0.7 mL/min. The peak fractions were collected and analyzed by Western blotting, SDS-PAGE and BN-PAGE 15%.

### 4.2. SDS-PAGE and BN-PAGE 15%

The analysis of 30 µg of proteins by BN-PAGE and SDS-PAGE 15% was performed according to standard protocols [39]. BN-PAGE measured the difference in electrophoretic migration and size between ETE, β-trypsin from bovine pancreas (Sigma-Aldrich) and bovine serum albumin monomer (Sigma-Aldrich), while a molecular marker Amersham ECL Rainbow (GE-Healthcare) was used in SDS-PAGE. The samples were fixed and stained in a mixture containing methanol, water, acetic acid (50:50:10 *v*/*v*) and Coomassie Brilliant Blue 0.25%.

### 4.3. Enzymatic Assays

The esterolytic activity of ETE and ETE/P186G against the synthetic substrate Boc-L-Glu-↓OPh was determined as previously reported for ETA and ETB [19,23,29]. Briefly, solutions were prepared with a total volume of 800 μL, containing ETE or ETE/P186G, each at a final concentration of 1 μg/mL, 1-4 dioxane (Sigma) 2% (*v*/*v*) and variable Boc-L-Glu-↓OPh concentrations (1.0, 5.0, 10.0, 15.0 and 20.0 mM) dissolved in HEPES 50 mM pH 7.4 buffer. The reaction progress was followed by measuring the absorbance at λ = 270 nm and *T* = 37 °C every 15 s for 20 min with a Cary UV-Vis Compact Peltier (Agilent) spectrophotometer. Each condition was assayed in triplicate. Initial velocities were estimated by finding the slopes of absorbance vs. time data through linear regression. Then, the kinetic parameters for ETE and ETE/P186G were estimated using the Hanes–Woolf linearization method [33]. Before the assays, Boc-L-Glu-↓OPh was synthesized as described in Text S2 and Figure S12.

### 4.4. Crystallization, Data Collection, Processing and Structure Determination

Crystals were obtained by hanging vapor diffusion from a buffer solution containing 30 mg/mL ETE, 20 mM Tris-HCl, pH 7.0 and 100 mM NaCl, which was equilibrated against a reservoir solution (4 M sodium formate, pH 6.0). Protein crystals were then frozen in a nitrogen gas stream at 100 K and diffracted at the W01B-MX2 beamline at the Brazilian Synchrotron Laboratory (LNLS, Campinas, Brazil). The wavelength of the radiation source was set to 1.458 Å and a Pilatus 2M detector was used to record the diffraction intensities. The crystal was exposed for 2 s per 0.1 degree of rotation and a total of 3600 images were collected. The data was indexed using the XDS package [40], and the structure was resolved by molecular replacement employing the atomic coordinates of another crystal structure of ETE (=ETD-like) (PDB: 5C2Z). Model refinement was performed through cycles of REFMAC5 [41] in the CCP4 software, followed by visual inspection of the electron density maps and manual reconstruction with WinCoot [42]. The validation of the new structure was carried out at the MolProbity server [43] and the atomic coordinates and properties of the crystal were deposited in the PDB with the code 8DAX.

### 4.5. Small-Angle X-ray Scattering Experiments

SAXS experiments were performed to evaluate the structural properties of ETE in solution close to the physiological conditions in terms of salt concentration and pH. The tests were conducted using the Xeuss 2.0 benchtop SAXS system from the XENOCS company, equipped with a Xenocs Genix 3D X-ray source with a copper anode that produces a beam with wavelength λ = 1.5419 Å. The collimation system, composed of a monochromator mirror and a set of scatterless slits, produces a beam of approximately 0.7 × 0.7 mm^2^. The system uses a Pilatus 300K Dectris detector for all measurements performed with samples under vacuum conditions. The sample–detector distance was calibrated through measurements of a silver behenate (AgBeh) standard that has peaks in the SAXS region providing a sample–detector distance of 1193 ± 8 mm. For measurement, 100 µL volume of ETE was used at a concentration of 5 mg/mL in 20 mM MES pH 7.0 and 150 mM NaCl. SAXS data were obtained from 7 cycles with run exposure times of 1800 s at a constant temperature of 25 °C. The 2D SAXS images were integrated with the Fit2D program [44]. Data integrations were processed and normalized in absolute scale with the SUPERSAXS program [45]. The treated curves were analyzed with the ATSAS software package [46], allowing us to obtain distribution curves of paired distances and to analyze the presence of oligomeric states.

SAXS can provide additional information that allows us to know the conformations of the ETE homodimers that better fit the experimental intensity profiles. As shown in Figure S6, the ability of 22 models of ETE homodimers to reproduce the experimental SAXS curves was assessed. For each model, we separated the monomer and homodimer structures. To evaluate the presence of monomers, homodimers or even mixtures in the system, the program Oligomer was used [46], which assessed the goodness of fit through chi-squared (χ^2^) values and estimated the percentage of homodimers associated with each analyzed model. It was allowed to add a constant during data fitting. In principle, the homodimer models yielding theoretical intensity profiles with lower χ^2^ values with respect to the experimental curve are more likely to be close to the actual ETE conformation in solution [35].

### 4.6. Computational Approaches to Predict the Conformation of ETE Homodimers

Each monomer of the reported ETE crystal structure (PDB: 8DAX) was submitted for ab initio docking at the web servers of five different protein–protein docking algorithms, i.e., ClusPro (https://cluspro.bu.edu/, access: date 10 August 2021) [36], HDOCK (http://hdock.phys.hust.edu.cn/, access date: 13 August 2021) [47], HADDOCK2.4 (https://bianca.science.uu.nl/haddock2.4/, access date: 20 August 2021) [48], GalaxyTongDock (GalaxyWeb: http://galaxy.seoklab.org/, access date: 18 August 2021) [49,50] and LZerD (https://lzerd.kiharalab.org/, access date: 21 August 2021) [51], in the above order and employing in each case the default parameters. Of note, HADDOCK2.4 docking simulations were restricted to a protein region that was predicted to contain interface-forming residues according to the CPORT (https://alcazar.science.uu.nl/services/CPORT/, access date: 25 August 2021) [52]. LZerD docking poses were generated using two different approaches, one of them involving C_2_-symmetric docking and the other one not assuming a specific symmetry. Moreover, homology-based models of the homodimer structure were obtained at the GalaxyWeb site [50,53]. The best nonredundant poses, i.e., those with pairwise RMSDs relative to their heavy atoms > 4 Å among the ten top-ranked solutions generated by each docking algorithm, were selected for refinement steps consisting of MD simulations, as well as MM-GBSA and US free energy calculations, in order to predict the most likely conformation of ETE dimers (Figure S6). SAXS was employed as an additional criterion for pose selection. The models yielding lower χ^2^ values and, at the same time, having favorable US free energy values were selected for the next steps, which consisted of 1 μs-long MD simulations and additional free energy calculations (Figure S6).

The central structure collected from the 1 μs MD simulation of the selected model was subjected to further bioinformatic analyses to assess whether it corresponds to a biological assembly or not, using PISA (https://www.ebi.ac.uk/msd-srv/prot_int/cgi-bin/piserver, access date: 9 September 2021) [26] and PRODIGY-CRYSTAL (https://bianca.science.uu.nl/prodigy/cryst, access date: 9 September 2021) [27] web servers. Moreover, SPPIDER was employed to predict whether dimer conformations contained IFRs [37]. SPPIDER makes use of solvent accessibility, amino acid residue conservation, charge conservation, amino acid residue size conservation, contacts and hydrophobicity in order to feed a neural network to classify residues on the interface or free surface. The model finally proposed as the most reliable conformation of ETE in solution was the one that passed all the steps of the devised protocol.

During the execution of this work, some reports showed that AlphaFold2-based approaches can predict the structure of protein–protein complexes with higher accuracy than protein–protein docking algorithms [54,55]. Therefore, we decided to assess in parallel the performance of these new approaches when applied to the prediction of the ETE homodimer structure. We employed for that purpose the AlphaFold-multimer [54] version implemented within the ColabFold open-source software [56]. As before, the top-ranked model was subjected to 100 ns MD simulations and subsequent free energy calculations. Moreover, the ability of this model to fit the SAXS data was assessed. 

### 4.7. Molecular Dynamics Simulations

ETs were protonated at the H++ web server (http://newbiophysics.cs.vt.edu/H++/, access date: 16 July 2021) by setting the pH = 7.4 [57]. Each complex was then solvated in an octahedral box with edges placed at least 10 Å away from the protein surface using *tleap* of Amber20 [58]. The simulation box was then filled with TIP3P waters, and sufficient counterions (Na^+^ or Cl^-^) were added to neutralize the net charge. The protein parameters were derived from Amber ff14SB force-field [59]. All MD simulations were run with *pmemd.cuda* of Amber 20 [58,60]. More details about the MD simulation setup can be found elsewhere [61].

Each solvated system was subjected to energy minimization (EM) followed by an NVT heating and NPT equilibration, each run for 500 ps in the presence of harmonic restraints acting on the protein heavy atoms (k *=* 10 kcal∙mol^−1^∙Å^−2^), to reach a temperature of 298 K and a pressure of 1 bar. Then, the harmonic restraints were gradually released from 8 to 2 kcal∙mol^−1^∙Å^−2^, with a 2 kcal∙mol^−1^∙Å^−2^ stride, in four 500 ps NPT simulations. All production runs were conducted using hydrogen mass repartitioning (HMR) in order to increase the time step from 2 to 4 fs [62].

### 4.8. MM-GBSA Free Energy Calculations

MM-GBSA free energy calculations were calculated for all the simulated ETE homodimers with the MMPBSA.py program of Amber 20 [58,63]. The generated 100 ns/1 μs trajectories were desolvated, and the frames collected after 10 ns/100 ns were used to calculate the corresponding Δ*G_eff_* values, which do not contain the configurational entropy component. The trajectories of the free monomers were extracted from those of the simulated complexes following the so-called single trajectory approach. The GB^OBC^ (*igb =* 2) implicit solvation model was employed to estimate the polar solvation free energies [58,64]. The internal and external dielectric constants were taken as default and the salt concentration was set to 0.1 M. The atomic radii were derived from the mbondi2 set, as recommended [58]. Moreover, per-residue free energy decomposition was carried out for the ETE homodimer with the GB^OBC^ model using MMPBSA.py [63] in order to predict the hotspot residues at the PPI.

### 4.9. Umbrella Sampling Free Energy Calculations

US is a robust approach to estimating binding free energies (∆*G_bind_*) [65] and thus was employed to predict more accurately the most favorable ETE homodimer conformation. As a result, PMFs associated with the separation of the ETE monomers from different initial ETE homodimer conformations along the *z* Cartesian axis were determined. Briefly, different ETE homodimer conformations were carefully oriented, and the monomers were separated along the *z* axis by displacing the second chain from −2 to 20 Å relative to its equilibrium position in the starting conformation. In total, 23 windows separated by 1 Å were generated and the proteins were attached by means of a special set of restraints to three dummy atoms fixed within the simulation box. This prevented the translation and rotation motions of the ETE chains relative to their centers of mass during the MD simulations carried out for each window [66]. Harmonic potentials were also applied to keep the second protein chain at the corresponding restraint equilibrium position within each window.

Similarly, US free energy calculations were performed to determine the PMF associated with the rotation around the N-Cα-C-O dihedral (ξ) of residue P186 of ETE. Windows were created every 5° from −180° to 175° to span the full rotation of the dihedral, and harmonic restraints were employed to keep the dihedral fluctuating around each pre-established equilibrium position. In all cases, PMFs were obtained by combining the results from all the simulated windows using the weighted histogram analysis (WHAM) [67]. Details of the US protocols employed here are included in Text S3. We followed identical procedures to calculate the PMFs associated with the rotation of N-Cα-C-O dihedrals of residue G186 of ETE/P186G (modeled from wild-type structure PDB 8DAX using Pymol mutagenesis plugging [32]) and residue G166 of V8 protease from *S. aureus* (PDB: 2O8L). Moreover, as a control, we obtained PMF for the rotation around the peptide bond (CA-C-N-CA dihedral) of the dipeptide L-alanyl-L-proline in solution at 313.15 K for which experimental data is available [28]. This allowed us to assess the accuracy of the US free energy calculation protocol. Details of the US protocols employed here are included in Text S3.

### 4.10. Trajectory Analyses

RMSDs and interatomic distances along the trajectories were calculated using the *rms* and *distance* commands, respectively, of *cpptraj* module of Amber20. Trajectory clustering was performed with the *cluster* command of *cpptraj* using the average linkage algorithm [58,68]. The RMSD with respect to the heavy atoms of the interface residues, defined by a 4 Å cut-off from each interacting chain, was set as a metric for the clustering analysis. As a rule of thumb, five central structures were generated per trajectory, and the one corresponding to the largest cluster was chosen for structural representation or as the starting structure for US free energy calculations. H-bonds were determined using *hbond* of *cpptraj* by setting a donor–acceptor upper cut-off distance of 3.5 Å and a donor–hydrogen–acceptor lower cut-off angle of 120°.

## 5. Conclusions

In the present work, we have presented results suggesting that the formation of homodimers may play a key role in controlling the enzymatic activity of ETE and, potentially, the remaining ETs. Moreover, we found that the flipped conformation of residue *192*, which has long been considered as the key structural feature of the ETs underlying their fine-tuned activity, does not seem to be as relevant to this process, at least for ETE. A better understanding of the exfoliative mechanism of the ETs will be valuable for the design of molecules with the capacity to modulate the enzymatic activity of these enzymes and thus prevent the degradation of their natural substrate Dsg1.

## Figures and Tables

**Table 1:** Crystallographic data processing and refinement statistics of ETE PDB structure 8DAX.

Data Collection	ETE
Wavelength (Å)	1.459
Resolution range (Å)	43.71–1.61 (1.67–1.61)
Space symmetry group	P 4(3) 2(1) 2
Cell dimensions:	
a, b, c (Å)	97.74, 97.74, 116.65
α, β, γ (º)	90.00, 90.00, 90.00
Total reflections	1,847,482 (175,190)
Unique reflections	72,995 (7181)
Multiplicity	25.3 (24.4)
Completeness range (%)	99.94 (99.85)
Mean I/σI	24.66 (1.07)
R-merge	0.076 (2.829)
Rmeas	0.077 (2.889)
Correlation coefficient ½	1 (0.52)
R_(work)_/R_(free)_	0.2206/0.2523
Number of protein chains in AU	2
Solvent water molecules	145
R.M.S:	
Bond lengths (Å)	0.015
Bond angles (º)	2.00
Ramachandran analysis:	
Favored (%)	95.33%
Allowed (%)	4.27%
Disallowed (%)	0.41%
Average B-factor (Å^2^)	36.50
PDB accession code	8DAX

## Data Availability

The reported structure of ETE (PDB: 8DAX) can be found at https://www.rcsb.org/ (accessed date: 25 August 2022). Other data regarding this work can be requested to the corresponding author.

## Supplementary Materials

The following supporting information can be downloaded at: https://www.mdpi.com/article/10.3390/ijms23179857/s1. More detail, refer to Refs. [17,28,32,34,58,62,65,66,69,70,71,72,73,74,75].

## References

[1] Bukowski M., Wladyka B., Dubin G. (2010). Exfoliative toxins of *Staphylococcus aureus*. Toxins.

[2] Mariutti R.B., Tartaglia N.R., Le Loir Y., Nishifuji K., Enany S., Crotty Alexander L.E. (2017). Exfoliative toxins of *Staphylococcus aureus*. The Rise of Virulence and Resistance in Staphylococcus aureus.

[3] Amagai M., Yamaguchi T., Hanakawa Y., Nishifuji K., Sugai M., Stanley J.R. (2002). Staphylococcal exfoliative toxin B specifically cleaves desmoglein 1. J. Investig. Dermatol..

[4] Hanakawa Y., Schechter N.M., Lin C., Nishifuji K., Amagai M., Stanley J.R. (2004). Enzymatic and molecular characteristics of the efficiency and specificity of exfoliative toxin cleavage of desmoglein 1. J. Biol. Chem..

[5] Hanakawa Y., Selwood T., Woo D., Lin C., Schechter N.M., Stanley J.R. (2003). Calcium-dependent conformation of desmoglein 1 is required for its cleavage by exfoliative toxin. J. Investig. Dermatol..

[6] O’Toole P.W., Foster T.J. (1987). Nucleotide sequence of the epidermolytic toxin A gene of *Staphylococcus aureus*. J. Bacteriol..

[7] Lee C.Y., Schmidt J.J., Johnson-Winegar A.D., Spero L., Iandolo J.J. (1987). Sequence determination and comparison of the exfoliative toxin A and toxin B genes from *Staphylococcus aureus*. J. Bacteriol..

[8] Yamaguchi T., Nishifuji K., Sasaki M., Fudaba Y., Aepfelbacher M., Takata T., Ohara M., Komatsuzawa H., Amagai M., Sugai M. (2002). Identification of the *Staphylococcus aureus* etd pathogenicity island which encodes a novel exfoliative toxin, ETD, and EDIN-B. Infect. Immun..

[9] Le Marechal C., Jardin J., Jan G., Even S., Pulido C., Guibert J.M., Hernandez D., Francois P., Schrenzel J., Demon D. (2011). *Staphylococcus aureus* seroproteomes discriminate ruminant isolates causing mild or severe mastitis. Vet. Res..

[10] Le Marechal C., Seyffert N., Jardin J., Hernandez D., Jan G., Rault L., Azevedo V., Francois P., Schrenzel J., van de Guchte M. (2011). Molecular basis of virulence in *Staphylococcus aureus* mastitis. PLoS ONE.

[11] Kapral F.A., Miller M.M. (1971). Product of *Staphylococcus aureus* responsible for the scalded-skin syndrome. Infect. Immun..

[12] Arbuthnott J.P., Kent J., Lyell A., Gemmell C.G. (1971). Toxic epidermal necrolysis produced by an extracellular product of *Staphylococcus aureus*. Br. J. Derm..

[13] Melish M.E., Glasgow L.A. (1970). The staphylococcal scalded-skin syndrome. N. Engl. J. Med..

[14] Imanishi I., Nicolas A., Caetano A.B., Castro T.L.P., Tartaglia N.R., Mariutti R., Guedon E., Even S., Berkova N., Arni R.K. (2019). Exfoliative toxin E, a new *Staphylococcus aureus* virulence factor with host-specific activity. Sci. Rep..

[15] Wegener H.C., Andresen L.O., Bille-Hansen V. (1993). Staphylococcus hyicus virulence in relation to exudative epidermitis in pigs. Can. J. Vet. Res..

[16] Chen S., Wang Y., Chen F., Yang H., Gan M., Zheng S.J. (2007). A highly pathogenic strain of *Staphylococcus sciuri* caused fatal exudative epidermitis in piglets. PLoS ONE.

[17] Li H., Wang Y., Ding L., Zheng S.J. (2011). *Staphylococcus sciuri* exfoliative toxin C (ExhC) is a necrosis-inducer for mammalian cells. PLoS ONE.

[18] Demidyuk I.V., Chukhontseva K.N., Kostrov S.V. (2017). Glutamyl Endopeptidases: The Puzzle of Substrate Specificity. Acta Nat..

[19] Cavarelli J., Prevost G., Bourguet W., Moulinier L., Chevrier B., Delagoutte B., Bilwes A., Mourey L., Rifai S., Piemont Y. (1997). The structure of *Staphylococcus aureus* epidermolytic toxin A, an atypic serine protease, at 1.7 A resolution. Structure.

[20] Vath G.M., Earhart C.A., Rago J.V., Kim M.H., Bohach G.A., Schlievert P.M., Ohlendorf D.H. (1997). The structure of the superantigen exfoliative toxin A suggests a novel regulation as a serine protease. Biochemistry.

[21] Papageorgiou A.C., Plano L.R., Collins C.M., Acharya K.R. (2000). Structural similarities and differences in *Staphylococcus aureus* exfoliative toxins A and B as revealed by their crystal structures. Protein Sci..

[22] Iyori K., Futagawa-Saito K., Hisatsune J., Yamamoto M., Sekiguchi M., Ide K., Son W.G., Olivry T., Sugai M., Fukuyasu T. (2011). Staphylococcus pseudintermedius exfoliative toxin EXI selectively digests canine desmoglein 1 and causes subcorneal clefts in canine epidermis. Vet. Derm..

[23] Bailey C.J., Redpath M.B. (1992). The esterolytic activity of epidermolytic toxins. Biochem. J..

[24] Rago J.V., Vath G.M., Tripp T.J., Bohach G.A., Ohlendorf D.H., Schlievert P.M. (2000). Staphylococcal exfoliative toxins cleave alpha- and beta-melanocyte-stimulating hormones. Infect. Immun..

[25] Mariutti R.B., Souza T.A., Ullah A., Caruso I.P., de Moraes F.R., Zanphorlin L.M., Tartaglia N.R., Seyffert N., Azevedo V.A., Le Loir Y. (2015). Crystal structure of Staphylococcus aureus exfoliative toxin D-like protein: Structural basis for the high specificity of exfoliative toxins. Biochem Biophys Res Commun.

[26] Krissinel E., Henrick K. (2007). Inference of macromolecular assemblies from crystalline state. J. Mol. Biol..

[27] Jimenez-Garcia B., Elez K., Koukos P.I., Bonvin A.M., Vangone A. (2019). PRODIGY-crystal: A web-tool for classification of biological interfaces in protein complexes. Bioinformatics.

[28] Shibukawa M., Miyake A., Eda S., Saito S. (2015). Determination of the cis-trans isomerization barriers of L-alanyl-L-proline in aqueous solutions and at water/hydrophobic interfaces by on-line temperature-jump relaxation HPLC and dynamic on-column reaction HPLC. Anal. Chem..

[29] Rago J.V., Vath G.M., Bohach G.A., Ohlendorf D.H., Schlievert P.M. (2000). Mutational analysis of the superantigen staphylococcal exfoliative toxin A (ETA). J. Immunol..

[30] Kawalec M., Potempa J., Moon J.L., Travis J., Murray B.E. (2005). Molecular diversity of a putative virulence factor: Purification and characterization of isoforms of an extracellular serine glutamyl endopeptidase of Enterococcus faecalis with different enzymatic activities. J. Bacteriol..

[31] Meijers R., Blagova E.V., Levdikov V.M., Rudenskaya G.N., Chestukhina G.G., Akimkina T.V., Kostrov S.V., Lamzin V.S., Kuranova I.P. (2004). The crystal structure of glutamyl endopeptidase from Bacillus intermedius reveals a structural link between zymogen activation and charge compensation. Biochemistry.

[32] DeLano W.L. (2002). PyMOL, 2.1.0.

[33] Hanes C.S. (1932). Studies on plant amylases: The effect of starch concentration upon the velocity of hydrolysis by the amylase of germinated barley. Biochem. J..

[34] Li H., Li X., Lu Y., Wang X., Zheng S.J. (2011). *Staphylococcus sciuri* exfoliative toxin C is a dimer that modulates macrophage functions. Can. J. Microbiol..

[35] Oliveira C.L.P., Chandasekaran A. (2011). Investigating macromolecular complexes in solution by small angle X-Ray scattering. Current Trends in X-Ray Crystallography.

[36] Kozakov D., Hall D.R., Xia B., Porter K.A., Padhorny D., Yueh C., Beglov D., Vajda S. (2017). The ClusPro web server for protein-protein docking. Nat. Protoc..

[37] Porollo A., Meller J. (2007). Prediction-based fingerprints of protein-protein interactions. Proteins.

[38] Gaber A., Pavsic M. (2021). Modeling and structure determination of homo-oligomeric proteins: An overview of challenges and current approaches. Int. J. Mol. Sci..

[39] Peters K., Braun H.P., Cramer R., Westermeier R. (2012). Comparative analyses of protein complexes by blue native DIGE. Difference Gel Electrophoresis (DIGE) Methods and Protocols.

[40] Kabsch W. (2010). XDSS. Acta Cryst. D Biol Cryst..

[41] Murshudov G.N., Skubak P., Lebedev A.A., Pannu N.S., Steiner R.A., Nicholls R.A., Winn M.D., Long F., Vagin A.A. (2011). REFMAC5 for the refinement of macromolecular crystal structures. Acta Cryst. D Biol. Cryst..

[42] Emsley P., Cowtan K. (2004). Coot: Model-building tools for molecular graphics. Acta Cryst. D Biol. Cryst..

[43] Chen V.B., Arendall W.B., Headd J.J., Keedy D.A., Immormino R.M., Kapral G.J., Murray L.W., Richardson J.S., Richardson D.C. (2010). MolProbity: All-atom structure validation for macromolecular crystallography. Acta Cryst. D Biol. Cryst..

[44] Hammersley A.P. (2016). FIT2D: A multi-purpose data reduction, analysis and visualization program. J. Appl. Cryst..

[45] Oliveira C.L.P., Vorup-Jensen T., Andersen C.B.F., Andersen G.R., Pedersen J.S., Gomez M., Nogales A., Garcia-Gutierrez M.C., Ezquerra T.A. (2009). Discovering new features of protein complexes structures by small-angle X-ray scattering. Applications of Synchrotron Light to Scattering and Diffraction in Materials and Life Sciences.

[46] Petoukhov M.V., Franke D., Shkumatov A.V., Tria G., Kikhney A.G., Gajda M., Gorba C., Mertens H.D., Konarev P.V., Svergun D.I. (2012). New developments in the ATSAS program package for small-angle scattering data analysis. J. Appl. Cryst..

[47] Yan Y., Tao H., He J., Huang S.Y. (2020). The HDOCK server for integrated protein-protein docking. Nat. Protoc..

[48] Dominguez C., Boelens R., Bonvin A.M. (2003). HADDOCK: A protein-protein docking approach based on biochemical or biophysical information. J. Am. Chem. Soc..

[49] Park T., Baek M., Lee H., Seok C. (2019). GalaxyTongDock: Symmetric and asymmetric ab initio protein-protein docking web server with improved energy parameters. J. Comput. Chem..

[50] Ko J., Park H., Heo L., Seok C. (2012). GalaxyWEB server for protein structure prediction and refinement. Nucleic. Acids Res..

[51] Christoffer C., Bharadwaj V., Luu R., Kihara D. (2021). LZerD protein-protein docking webserver enhanced with de novo structure prediction. Front. Mol. Biosci..

[52] de Vries S.J., Bonvin A.M. (2011). CPORT: A consensus interface predictor and its performance in prediction-driven docking with HADDOCK. PLoS ONE.

[53] Park T., Won J., Baek M., Seok C. (2021). GalaxyHeteromer: Protein heterodimer structure prediction by template-based and ab initio docking. Nucleic Acids Res..

[54] Evans R., O’Neill M., Pritzel A., Antropova N., Senior A., Green T. (2021). Protein complex prediction with AlphaFold-Multimer. Biorxiv [Preprint].

[55] Ghani U., Desta I., Jindal A., Khan O., Jones G., Kotelnikov S., Padhorny D., Vajda S., Kozakov D. (2021). Improved docking of protein models by a combination of alphafold2 and cluspro. Biorxiv [Preprint].

[56] Mirdita M., Schutze K., Moriwaki Y., Heo L., Ovchinnikov S., Steinegger M. (2022). ColabFold: Making protein folding accessible to all. Nat. Methods.

[57] Gordon J.C., Myers J.B., Folta T., Shoja V., Heath L.S., Onufriev A. (2005). H^++^: A server for estimating pKas and adding missing hydrogens to macromolecules. Nucleic Acids Res..

[58] Case D.A., Belfon K., Ben-Shalom I.Y., Brozell S.R., Cerutti D.S., Cheatham T.E., Cruzeiro V.W.D., Darden T.A., Duke R.E., Giambasu G. (2020). Amber 2020.

[59] Maier J.A., Martinez C., Kasavajhala K., Wickstrom L., Hauser K.E., Simmerling C. (2015). ff14SB: Improving the accuracy of protein side chain and backbone parameters from ff99SB. J. Chem. Theory Comput..

[60] Salomon-Ferrer R., Gotz A.W., Poole D., Le Grand S., Walker R.C. (2013). Routine microsecond molecular dynamics simulations with AMBER on GPUs. 2. Explicit solvent particle mesh ewald. J. Chem. Theory Comput..

[61] Hernandez Gonzalez J.E., Hernandez Alvarez L., Pascutti P.G., Valiente P.A. (2017). Predicting binding modes of reversible peptide-based inhibitors of falcipain-2 consistent with structure-activity relationships. Proteins.

[62] Hopkins C.W., Le Grand S., Walker R.C., Roitberg A.E. (2015). Long-time-step molecular dynamics through hydrogen mass repartitioning. J. Chem. Theory Comput..

[63] Miller B.R., McGee T.D., Swails J.M., Homeyer N., Gohlke H., Roitberg A.E. (2012). MMPBSA.py: An efficient program for end-state free energy calculations. J. Chem. Theory Comput..

[64] Onufriev A., Bashford D., Case D.A. (2004). Exploring protein native states and large-scale conformational changes with a modified generalized born model. Proteins.

[65] Kästner J. (2011). Umbrella sampling. Wiley Interdiscip. Rev. Comput. Mol. Sci..

[66] Heinzelmann G., Henriksen N.M., Gilson M.K. (2017). Attach-pull-release calculations of ligand binding and conformational changes on the first BRD4 bromodomain. J. Chem. Theory Comput..

[67] Kumar S., Rosenberg J.M., Bouzida D., Swendsen R.H., Kollman P.A. (1992). The weighted histogram analysis method for free-energy calculations on biomolecules. I. The method. J. Comput. Chem..

[68] Shao J., Tanner S.W., Thompson N., Cheatham T.E. (2007). Clustering molecular dynamics trajectories: 1. Characterizing the performance of different clustering algorithms. J. Chem. Theory Comput..

[69] Hong P., Koza S., Bouvier E.S. (2012). Size-Exclusion Chromatography for the Analysis of Protein Biotherapeutics and their Aggregates. J. Liq. Chromatogr. Relat. Technol..

[70] Madhusudhan M.S., Webb B.M., Marti-Renom M.A., Eswar N., Sali A. (2009). Alignment of multiple protein structures based on sequence and structure features. Protein Eng. Des. Sel..

[71] Larkin M.A., Blackshields G., Brown N.P., Chenna R., McGettigan P.A., McWilliam H., Valentin F., Wallace I.M., Wilm A., Lopez R. (2007). Clustal W and Clustal X version 2.0. Bioinformatics.

[72] Robin S., Zhu J., Galons H., Chuong P.-H., Claude J.R., Tomas A., Viossat B. (1995). A convenient asymmetric synthesis of thalidomide. Tetrahedron Asymmetry.

[73] Doudou S., Burton N.A., Henchman R.H. (2009). Standard Free Energy of Binding from a One-Dimensional Potential of Mean Force. J Chem Theory Comput.

[74] Abraham M.J., Murtola T., Schulz R., Páll S., Smith J.C., Hess B., Lindahl E. (2015). GROMACS: High performance molecular simulations through multi-level parallelism from laptops to supercomputers. SoftwareX.

[75] Hernandez Gonzalez J.E., Hernandez Alvarez L., Pascutti P.G., Leite V.B.P. (2019). Prediction of Noncompetitive Inhibitor Binding Mode Reveals Promising Site for Allosteric Modulation of Falcipain-2. J. Phys. Chem. B.

