# Supplementary material for: Staphylococcus aureus Exfoliative Toxin E, Oligomeric State and Flip of P186: Implications for Its Action Mechanism
Source: Int J Mol Sci. 2022 Aug 30;23(17):9857. doi: 10.3390/ijms23179857 (PMC9456352; doi:10.3390/ijms23179857)
Supplement: Supplementary file 1 [file ijms-23-09857-s001.zip › ijms-1871795-supplementary.pdf]

Supplemental Material

Staphylococcus aureus exfoliative toxin E, oligomeric state and flip of P186: implications for its action mechanism

Carolina Gismene [1,†], Jorge Enrique Hernández González [1,†], Angela Rocio Niño Santisteban [1], Andrey Fabricio Ziem Nascimento [2], Lucas dos Santos Cunha [3], Fábio Rogério de Moraes [1], Cristiano Luis Pinto de Oliveira [4], Caio C. Oliveira [3], Paola Jocelan Scarin Provazzi [5], Pedro Geraldo Pascutti [6], Raghuvir Krishnaswamy Arni [1,*] and Ricardo Barros Mariutti [1,*]

1 Multiuser Center for Biomolecular Innovation, IBILCE/UNESP, São José do Rio Preto 15054-000, Brazil
2 Brazilian Synchrotron Light Laboratory (LNLS), Brazilian Center for Research in Energy and Materials (CNPEM), Campinas 13083-970, Brazil
3 Institute of Chemistry, University of Campinas, Campinas 13083-970, Brazil
4 Instituto de Física, University of São Paulo, São Paulo 05314-970, Brazil; crislpo@if.usp.br (C.L.P.O)
5 Laboratory of Genomic Studies, Sao Paulo State University-UNESP, São José do Rio Preto 15054-000, Brazil
6 Laboratório de Modelagem e Dinâmica Molecular, Instituto de Biofísica Carlos Chagas Filho, Universidade Federal do Rio de Janeiro, Rio de Janeiro 21941-901, Brazil
* Correspondence: raghuvir.arni@unesp.br (R.K.A.); ricardomariutti@yahoo.com.br (R.B.M.)
† These authors contributed equally to this work.

TABLE OF CONTENTS

Supplementary Texts

Text S1. Size-exclusion chromatograms for ETE and ExhC……..……………..……….…………..……………………5

Text S2. Preparation of *N*-Tertbutyioxycarbonyl glutamic acid *O*-phenyl ester (Boc-L-Glu-Oph)…………………......17

Text S3. Details of the Umbrella sampling free energy calculation protocols…………………….……….…………..18

Supplementary Figures

Figure S1. Representation of the two chains in the asymmetric units in the available crystal structures of ETE..…..3

Figure S2. Fluctuation of P186 N-CA-C-O (ξ) and Y158 N-CA-CB-CG (χ_1) dihedral angles throughout 1 μs replicate MD simulations of ETE monomers in water……………………………………….………………..………..4

Figure S3. PMF for the rotation of L-alanyl-L-proline peptide bond……………….………………..………..….……..5

Figure S4. Comparison between ETE of *S. aureus* and ExhC of *S. sciuri*…………………..…................................6

Figure S5. Comparison between SEC chromatograms of ETE and ETE/P186G of *S. aureus* ……….………….……..7

Figure S6. Workflow for the prediction of the most stable ETE dimers in solution ………………..…………...……..8

Figure S7. Examples of SAXS intensity profile fits using three different homodimer models……………..…………..9

Figure S8. Models of ETE homodimer using ColabFold's AlphaFold-multimer version……………………………10

Figure S9. Structural alignment of different serine proteases shown at the level of their primary sequences......14

Figure S10. Structural alignment of different serine proteases……….…………..………………………..………..…15

Figure S11. Model of ETE in complex with Dsg1 EC3-EC4 linker and superposition with the predicted structure of ETE homodimer……………………………………………………………………...………………………..……….16

Figure S12. Synthesis of Boc-L-Glu-OPh……………………………………………………………………………..17

Supplementary Tables

Table S1. Analysis of the stability of putative ETE homodimers in the available crystal structures…………….…3

Table S2. Analyses conducted for the selected docking poses……………………..…….………………..…………….11

Table S3. Bioinformatic analysis of the stability of the predicted ETE homodimer..……………….………………...13

Table S4. Comparison of homodimer interfaces in the available crystal structures and the proposed model……..13

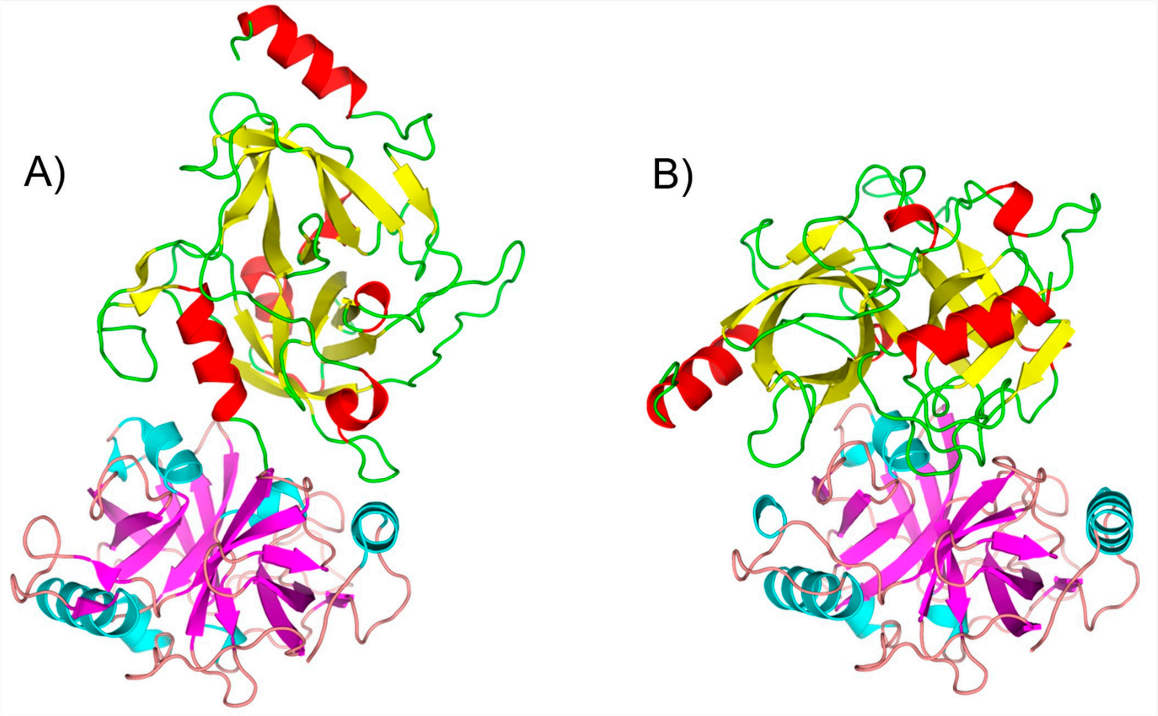

Figure S1. Representation of the two chains in the asymmetric units in the available crystal structures of ETE. A) PDB: 5C2Z. B) New crystal structure. Protein backbone is represented as cartoon and secondary structure elements are colored differently in each chain, i.e., α-helices, yellow/magenta for β-strands and green/salmon for loops in chains A/B. To facilitate the visual comparison of the structures, chain B is represented in the same orientation in both cases.

Table S1. Analysis of the stability of putative ETE homodimers in the available crystal structures.

**Structure**	Homodimer stability PISA	Homodimer stability PRODIGY-CRYSTAL	**$\Delta G_{eff,100ns}$ (kcal/mol)**	**$\Delta G_{PMF,1}$ (kcal/mol)**
5C2Z	Not stable	100% crystallographic	-7.2	-
8DAX	Not stable	65.6% crystallographic	-30.2	-0.3(0.1)

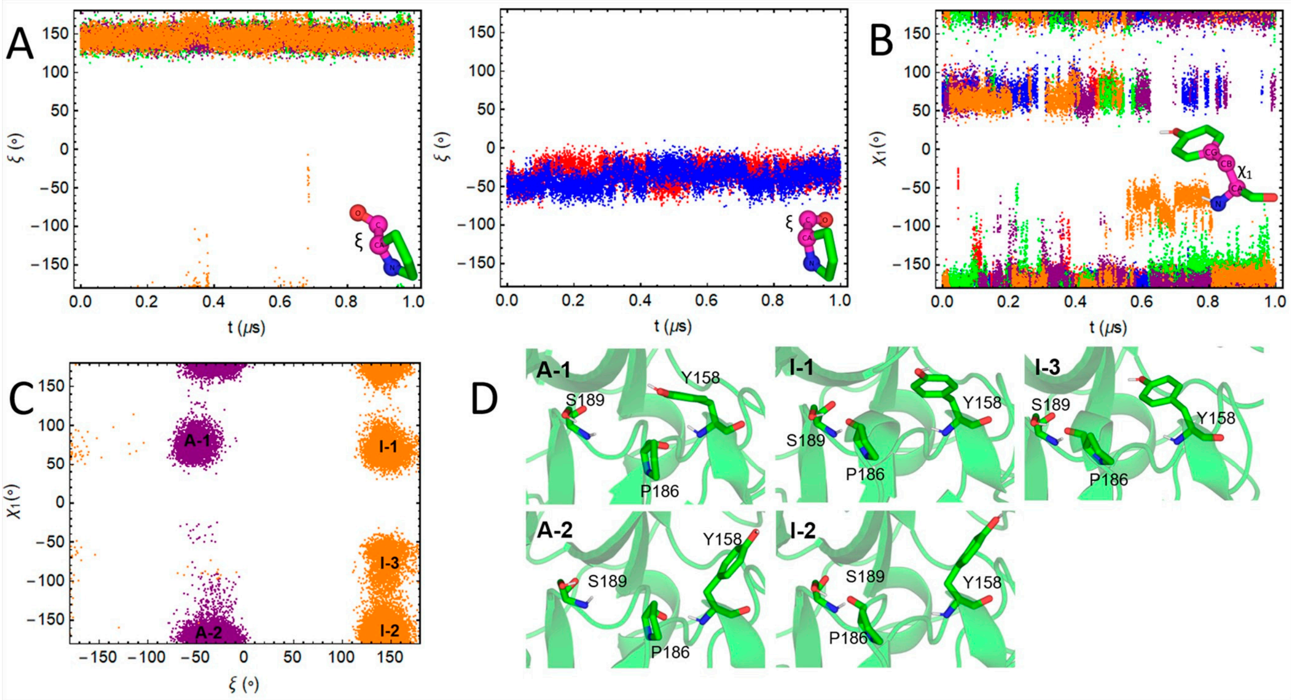

Figure S2. Fluctuation of P186 N-CA-C-O (ξ) and Y158 N-CA-CB-CG (χ_1) dihedral angles throughout 1 μs replicate MD simulations of ETE monomers in water. A) Time profiles of P186 ξ dihedral during five replicate MD simulations, three started from an ETE structure with an inactive conformation of P186 (graph on the left) and two, from an ETE structure with an active conformation of P186 (graph on the right). **B)** Time profiles of Y158 χ_1 dihedral along the five replicate MD simulations of ETE. **C)** Y158 χ_1 vs. P186 ξ distribution along the replicate MD simulations. The five main populations of dihedral pairs are labeled. Letter A and I indicate whether the P186 ξ dihedra corresponds to an active or inactive conformation. The accompanying numbers after the hyphen are employed to discriminate the different populations of Y158 χ_1 dihedral. **D)** Frames extracted from the MD trajectories representing each of the dihedral populations identified in C). In all cases, the profiles belonging to different replicate MD simulations are colored differently.

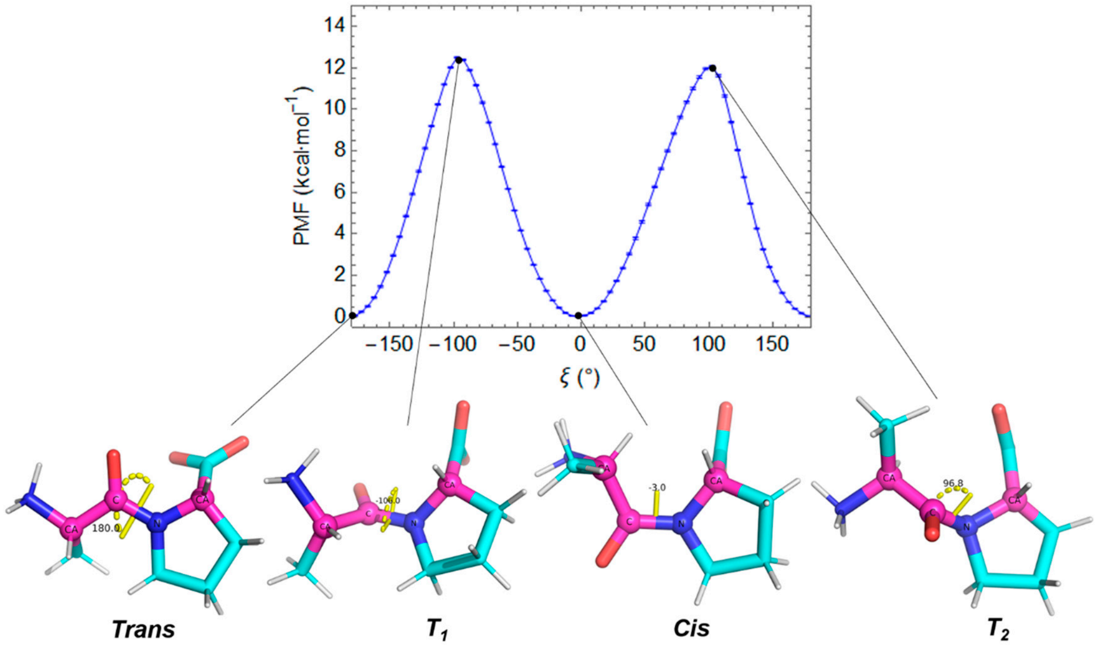

Figure S3. PMF for the rotation of L-alanyl-L-proline peptide bond. The structures of Ala-Pro conformations corresponding to free energy minima and transition states identified in the PMF are represented in the lower part of the figure. The local minima are the *trans* (CA-C-N-CA dihedral, ξ=±180º) and *cis* (ξ=0º) dipeptide isomers. The calculated free energy for the *cis*-*trans* isomerization ($\Delta G_{c\text{-}t}$) is 0.03±0.03 kcal/mol, which means that both states are isoenergetic, in agreement with the experimental $\Delta G_{c\text{-}t}$ value of -0.12±0.03 kcal/mol ($K_{c\text{-}t}$=1.21±0.06 at 313 K) [28]. The predicted free energies barriers associated with the *cis-trans* ($\Delta G^{\ddagger}_{c-t}$) and *trans-cis* ($\Delta G^{\ddagger}_{t-c}$) isomerization are 12.08±0.03 and 12.51±0.02 kcal/mol, respectively. The experimental energy barrier values for $\Delta G^{\ddagger}_{c-t}$ range from 18.2 to 20.8 kcal/mol, and for $\Delta G^{\ddagger}_{t-c}$, from 19.6 to 21.0 kcal/mol [28]. Therefore, the employed free energy method tends to underestimate the free energies of the T_1 and T_2 by 6 to 8 kcal/mol, probably as a result of inaccuracies inherent to modeling the molecular system with a classical force field.

Text S1. Size-exclusion chromatograms for ETE and ExhC

For the comparative analysis between ETE from *S. aureus* and ExhC from *S. sciuri*, experiments to obtain the latter protein were performed. *Escherichia coli* BL21(DE3) competent cells were transformed with pET28a(+)-ExhC expression vector. Then, we followed the same expression and purification protocol established for ETE from *S. aureus* (description in Protein expression and purification). ExhC was produced on a large-scale and high-purity (Figure S4B).

The chromatographic profiles of the ETE showed the main absorbance peak at elution volumes 10-12 ml, similar to the results observed for ExhC using the Superdex G75 10/300 GL column (GE) (Figure S4A). The large overlap between ExhC and ETE main elution peaks can be interpreted as a consequence of the high sequential and structural homology, as well as the identical oligomeric state

of both proteins. However, relatively small discrepancies in their elution volumes (Figure S4A) are expected because of the particular physicochemical characteristics of each ET [69]. In accordance with the SEC results, SDS-PAGE (Figure S4B) and BN-PAGE (Figure S4C) assays indicated that ETE and ExhC have similar sizes under denaturing (MW ~30 kDa) and native (MW ~56 kDa) conditions, respectively. This information reinforces that ETE is a dimer in solution, as previously proposed for ExhC [34].

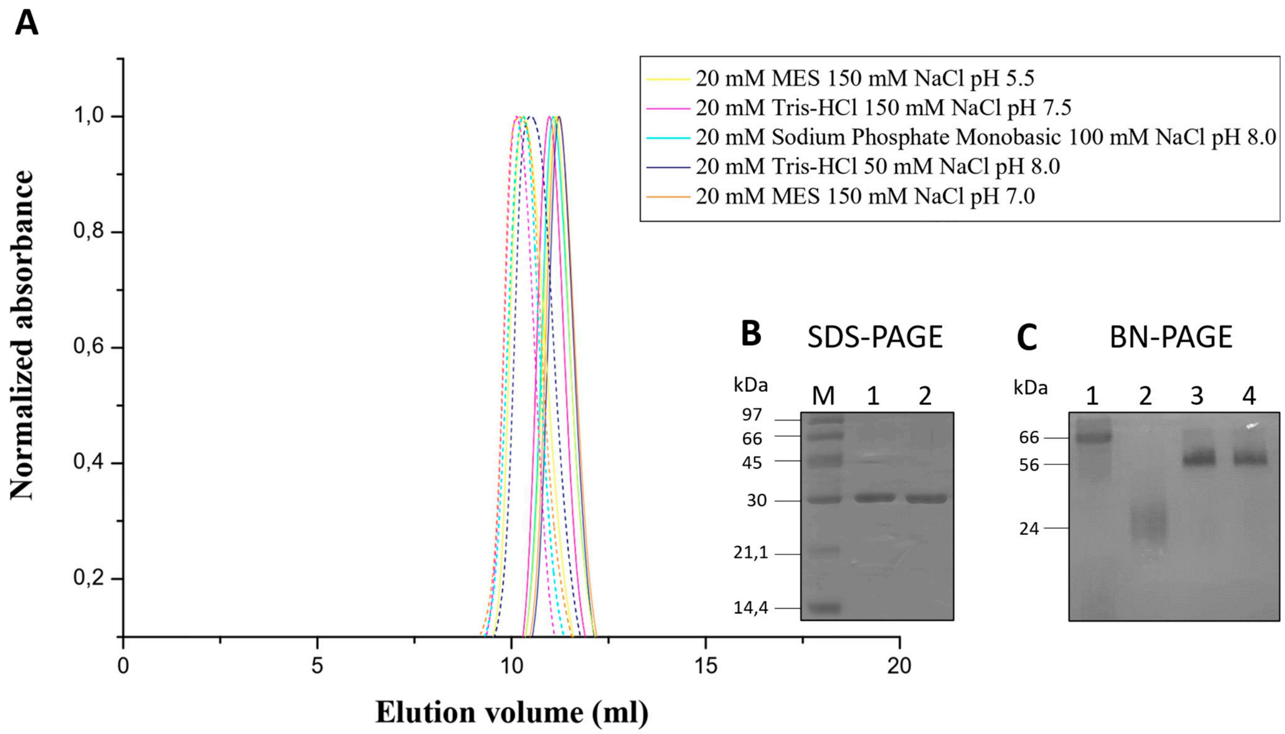

Figure S4. Comparison between ETE of *S. aureus* and ExhC of *S. sciuri*. A) SEC chromatograms for ETE (solid line) and ExhC (dash line) under different buffering conditions. Absorbance was measured at 230 nm in all cases, and normalized values are plotted. The overlap of peaks at ~10-12 ml indicates that ETE and ExhC elute in the same oligomeric state in all the tested conditions. **B)** SDS-PAGE of ETE (1) and ExhC (2) using the protein marker LMW-SDS Marker KIT – GE Healthcare (M). C) BN-PAGE of ETE (3) and ExhC (4) using BSA (1) and β-trypsin from bovine pancreas (2) as molecular weight markers. A running buffer containing 25 mM Tris pH 8.3 and 192 mM Glycine was employed for BN-PAGE.

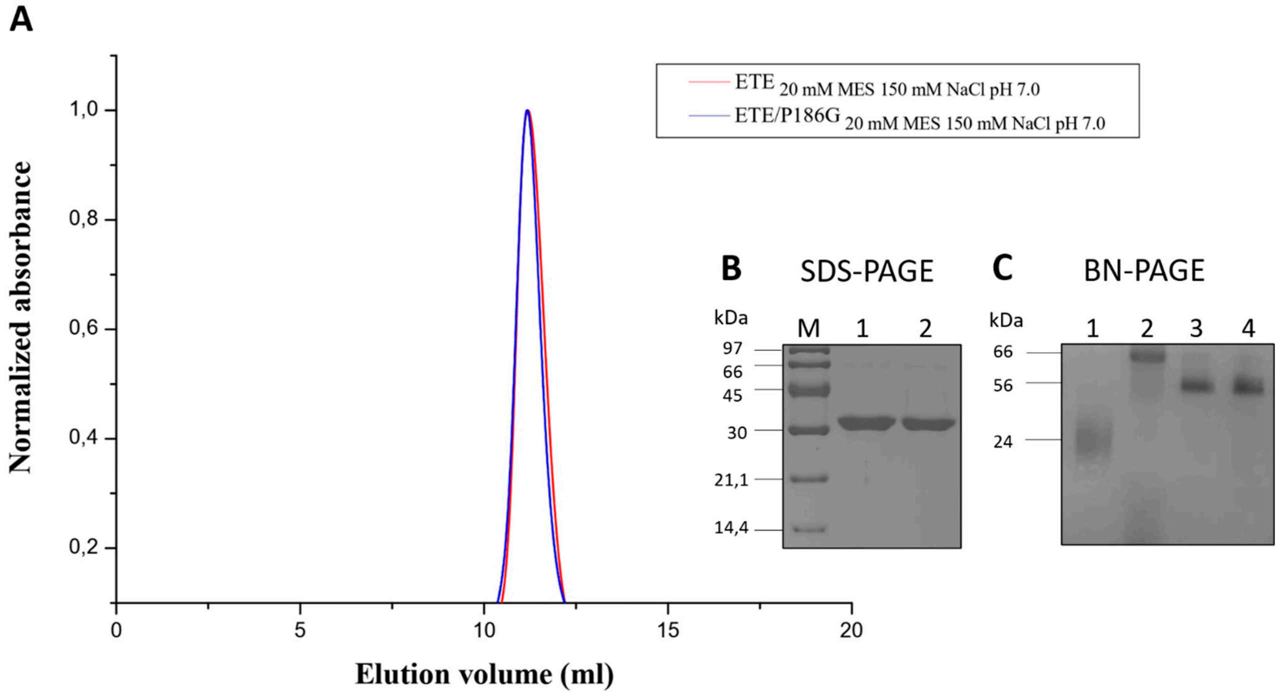

Figure S5. Comparison between SEC chromatograms of ETE and ETE/P186G of *S. aureus*. A) SEC chromatograms for ETE (red line) and ETE/P192G (blue line) in 20 mM MES pH 7.0 and 150 mM NaCl buffer. Absorbance was measured at 230 nm and normalized values are plotted. The overlap of peaks at ~10-12 ml indicates that ETE and ETE/P192G elute in the same oligomeric state in the tested condition. **B)** SDS-PAGE of ETE (1) and ETE/P192G (2) using the protein marker LMW-SDS Marker KIT – GE Healthcare (M). **C)** BN-PAGE of ETE (3) and ETE/P192G (4) using β-trypsin from bovine pancreas (1) and BSA (2) as molecular weight markers. A running buffer containing 25 mM Tris pH 8.3 and 192 mM Glycine was employed for BN-PAGE.

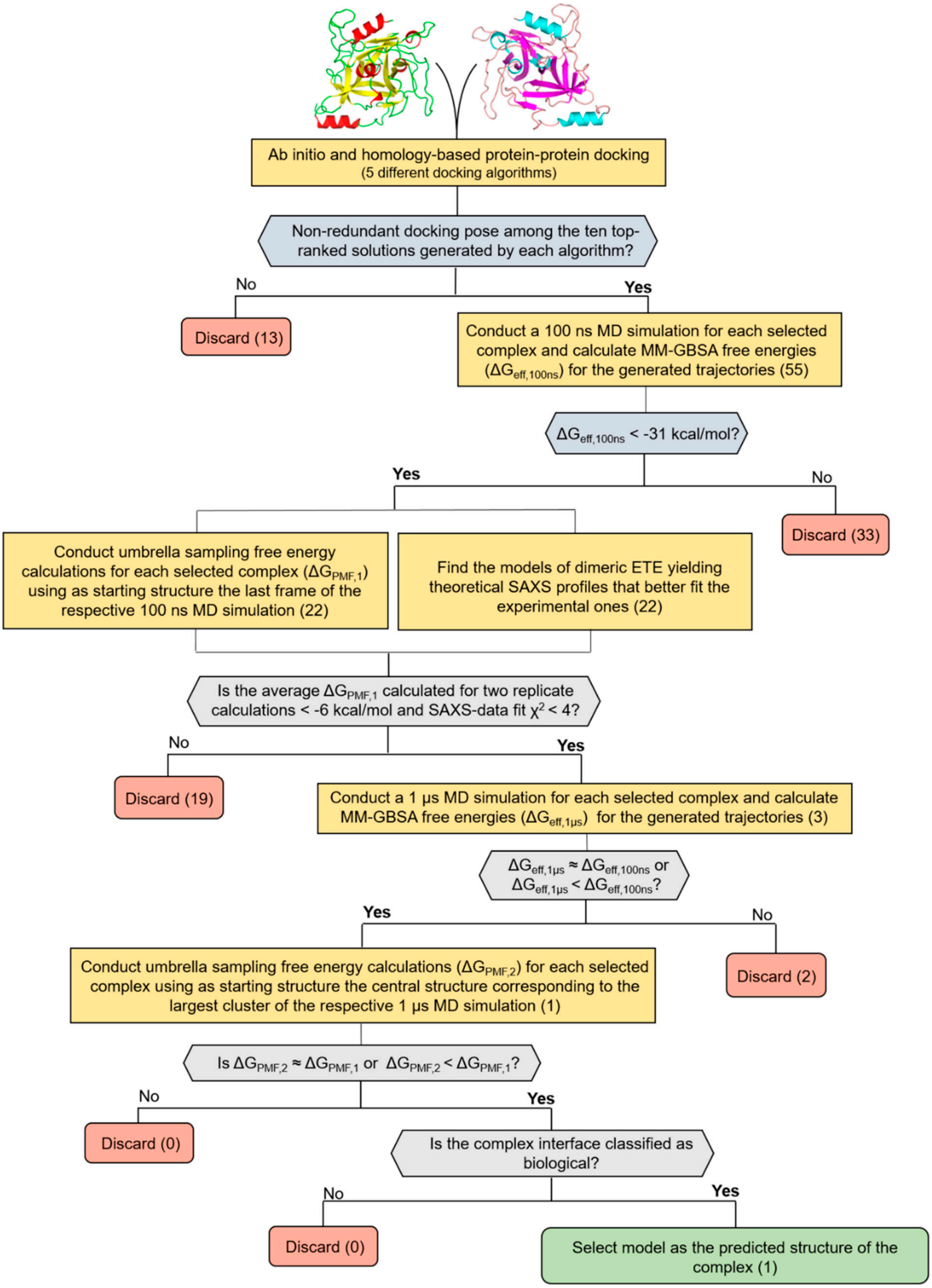

Figure S6. Workflow for the prediction of the most stable ETE dimers in solution. The number of models analyzed at every step is shown in parentheses. More details in Materials and Methods.

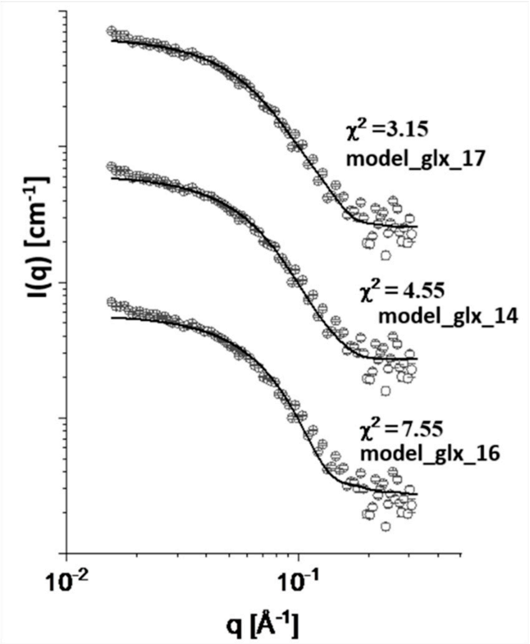

Figure S7. Examples of SAXS intensity profile fits using three different homodimer models. *I* and *q* stand or the intensity, expressed in arbitrary units, and the scattering vector, respectively. Models selected as examples will be discussed in the section below.

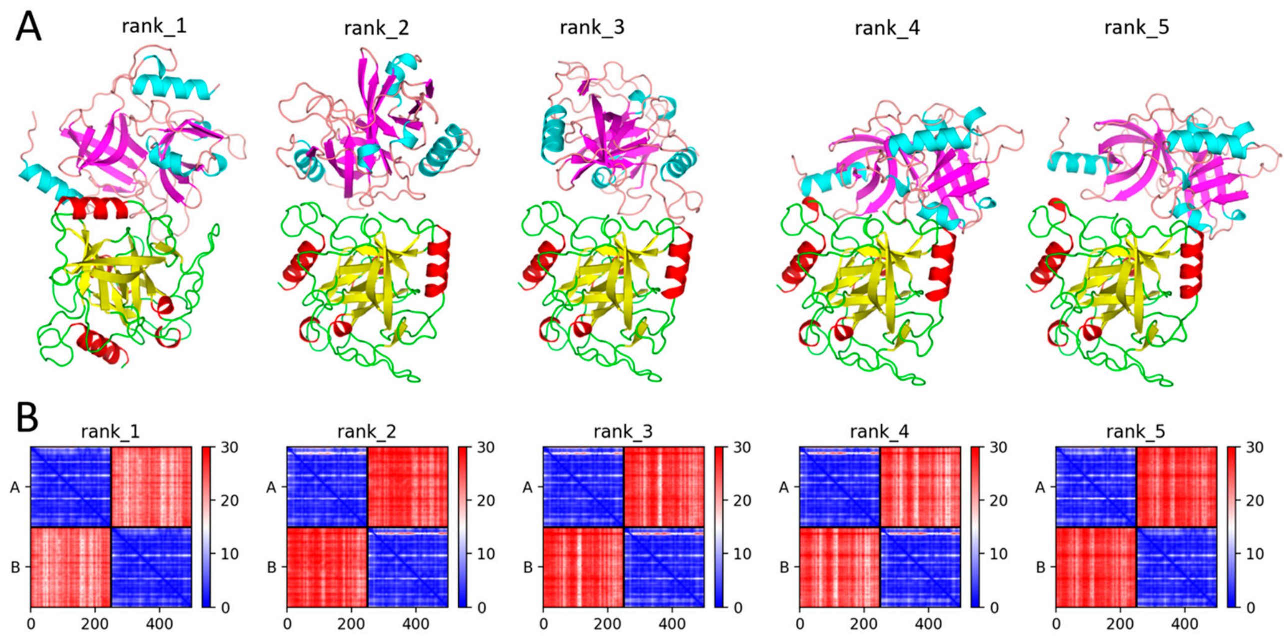

C

Model	$\Delta G_{eff,100ns}$ (kcal/mol)	SAXS χ^2	Dimer (%)
rank_1	-8.50	8.96	98.6

Figure S8. Models of ETE homodimer using ColabFold's AlphaFold-multimer version. A) Cartoon representation of five ETE homodimers. Each chain is colored differently. Models are ranked according to the average values of pLDDT metric. **B)** Predicted aligned error (PAE) matrices for the generated models. PAE is measured in Å. Low values indicate higher confidence in the positions of residues *x* and *y* are expected to be modeled correctly. The matrices indicate that AlphaFold2 correctly predicted the structures of the two monomers (blue squares), whereas low confidence is associated with the prediction of the homodimer conformation (red squares). **C)** Assessment of the top-ranked model accuracy through MM-GBSA free energy calculations based on 100 ns MD trajectories and SAXS-data fits. Both $\Delta G_{eff,100ns}$ and χ^2 values compared poorly with those estimated from several docking poses (Table S3), thus reinforcing that AlphaFold2-multimer did not performed well when predicting the homodimer conformation.

Table S2. Analyses conducted for the selected docking poses (to be continued).

Pose	Docking Algorithm	$\Delta G_{eff,100ns}$ (kcal/mol)	$\Delta G_{PMF,1}$ (kcal/mol)	SAXS χ^2	Dimer (%)	$\Delta G_{eff,1\mu s}$ (kcal/mol)	$\Delta G_{PMF,2}$ (kcal/mol)
**0**	ClusPro	-45.6	-10.4(1.1)	3.32	94.8	-56.2	-9.9(0.4)
1		-27.9					
2		-31.8	-7.1(0.8)	4.99	98.3		
3		-9.0					
4		-21.9					
5		-17.4					
6		-16.3					
7		-26.0					
8		-30.1					
9		-45.9	-7.4(1.0)	4.03	100		
1	HDOCK	-55.5	-10.3(0.4)	5.30	97.9		
2		-23.1					
3		-28.4					
4		-11.0					
5		-30.5					
6		-18.8					
7		-32.7	-2.9(1.0)	7.12	100		
**8**		-32.7	-5.2(1.8)	3.98	89.6		
9		-38.0	-6.7(0.2)	5.37	95.8		
**10**		-33.7	-6.4(0.3)	3.17	84.2	-2.76	
1	HADDOCK	-22.9					
2		-23.5					
3		-15.4					
4		-46.7	-6.1(2.7)	5.50	100		
5		-36.7	-9.0(1.3)	4.08	100		
6		-42.9	-3.6(0.6)	4.24	97.9		
7		-27.2					
8		-27.0					
9		-6.2					
10		-19.2					

Table S2. Analyses conducted for the selected docking poses.

Pose*	Docking Algorithm	Template PDB	$\Delta G_{eff,100ns}$ (kcal/mol)	$\Delta G_{PMF,1}$ (kcal/mol)	SAXS χ^2	Dimer (%)	$\Delta G_{eff,1\mu s}$ (kcal/mol)
1	GalaxyWeb Homodimer Homology Modeling	1P3E	-8.5				
2		4IW4	-1.4				
3		2HLC	-3.2				
4		1LVM	-21.1				
5		1FUJ	-11.8				
6		4WVP	-26.7				
**7**		4WVP	-52.4	-1.7(0.3)	3.33	84.5	
8		4NSV	0.24				
9	Galaxy TongDock		-37.8	-4.6(0.5)	5.54	100	
10			-46.0	-9.9(0.8)	4.66	100	
11			-18.8				
12			-29.0				
13			-31.3	-5.40(0.03)	5.00	100	
14			-35.6	-4.9(1.3)	4.55	96.9	
16			-33.1	-4.8(1.1)	7.55	100	
**17**			-58.9	-9.9(0.9)	3.15	91.7	-25.3
18			-10.9				
19			-33.5	-6.1(2.0)	5.30	95.8	
7	LZerD+C_2[a]		-13.0				
8			-21.6				
9			-7.8				
3	LZerD No C_2[b]		-39.3	-6.6(2.6)	5.32	100	
8			-34.9	-4.9(0.1)	5.00	99.0	
9			-35.5	-1.9(0.7)	5.00	89.6	
10			-20.1				

[a]Poses are numbered according to the ranking established by each docking algorithm. The lower the pose number, the higher the associated docking score. Poses labeled in bold correspond to the five models that yielded the lower chi-squared values when fitting the SAXS profiles.

[b]LZerD can be conducted by forcing the complexes to display C_2 symmetry or not.

Table S3. Bioinformatic analysis of the stability of the predicted ETE homodimer.

Structure	PISA	PRODIGY CRYSTAL	SPPIDER IFR[a]
ETE_2-CP-0	Stable (ΔG_{diss}=3.1 kcal/mol)[b]	60.4% biological (ΔG_{bind}=-8 kcal/mol)[c]	G89, L91, V92 (chain A) L91, V92 (chain B)

[a]Interface forming residues predicted by SPPIDER.
[b]Free energy of dissociation of the homodimer. A positive value indicates a stable oligomer.
[c]Free energy of binding of the homodimer. In this case, a negative value corresponds to a stable oligomer. In principle, $\Delta G_{bind} = -\Delta G_{diss}$

Table S4. Comparison of homodimer interfaces in the available crystal structures and the proposed model

Structure	Residues chain A[a]	Residues chain B[a]	Buried area (Å^2)[b]	RMSD (Å)[c]
5C2Z	K17, T18, K48, H66, R69, L70, E107, P136, S189, S205, G206, K207	E103, E104, I105, Y126, G127, D132, K231, T246, E248, Q249	542.5	0
8DAX	K48, T49, H66, I67, R69, L70, E72, E107, A108, G112, G113, Y158, N159, T177, P186, S189, G206, K207, G208, G209, Q210, F215, S226, Y227	K48, H66, R69, L70, E72, A108, G111, G112, G113, T177, P186, S189, S205, G206, K207, G209, Q210, F215, S226, Y227	1073.3	13.08
ETE_2-CP-0	K17, T18, R19, S21, F44, K48, T49, I50, R86, D87, G89, S90, L91, V92, Y158, N159, T160, S161, T162, Y183	K17, T18, R19, S21, F44, G47, K48, I50, R86, E88, G89, S90, L91, V92, Y158, N159, T160, S161, T162, H163, Y183, E185	1064.8	18.26, 22.21

[a]For comparison purposes, all residues appear in sequential order, as in PDB: 8DAX. A 4 Å cutoff was used to define the interface residues.
[b]Buried area of the complex interface was calculated with PISA.
[c]Heavy atom RMSD values between the homodimer conformations. The ETE structure 5C2Z was taken as reference for the calculation of all RMSD values, except for the second value of ETE_2-CP-0, which was determined using the structure 8DAX as reference.

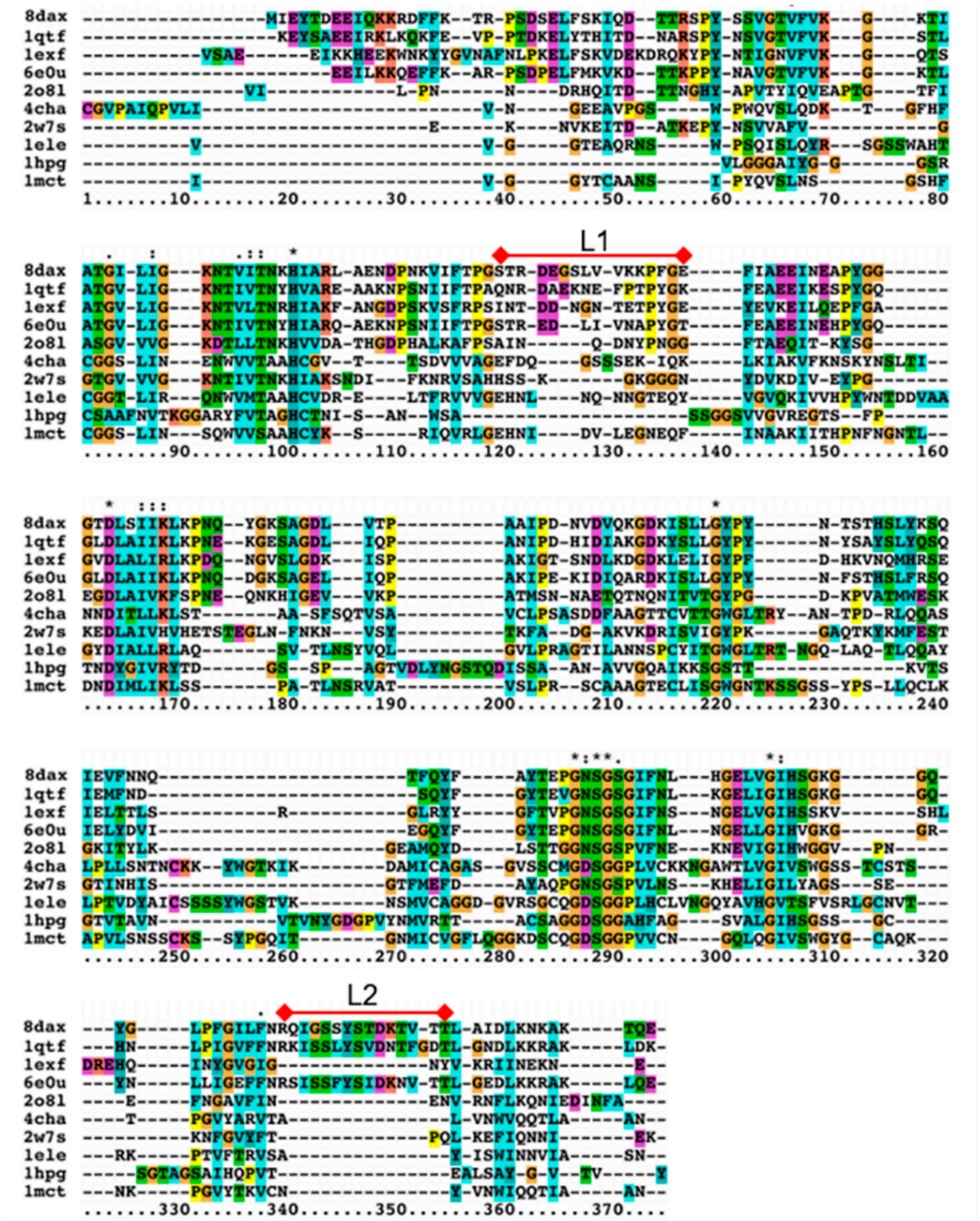

Figure S9. Structural alignment of different serine proteases shown at the level of their primary sequences. ETE (8DAX), ETB (1QTF), ETA (1EXF), EXI (6E0U), V8 protease (2O8L), Bovine chymotrypsin (4CHA), SP1A (2W7S), porcine pancreatic elastase (1ELE), Glu-SGP (1HPG) and bovine trypsin (1MCT) were structurally aligned at https://modbase.compbio.ucsf.edu/salign/ [70]. ClustalX2 [71] was used to visualize the alignment. Loops L1 and L2 are highlighted.

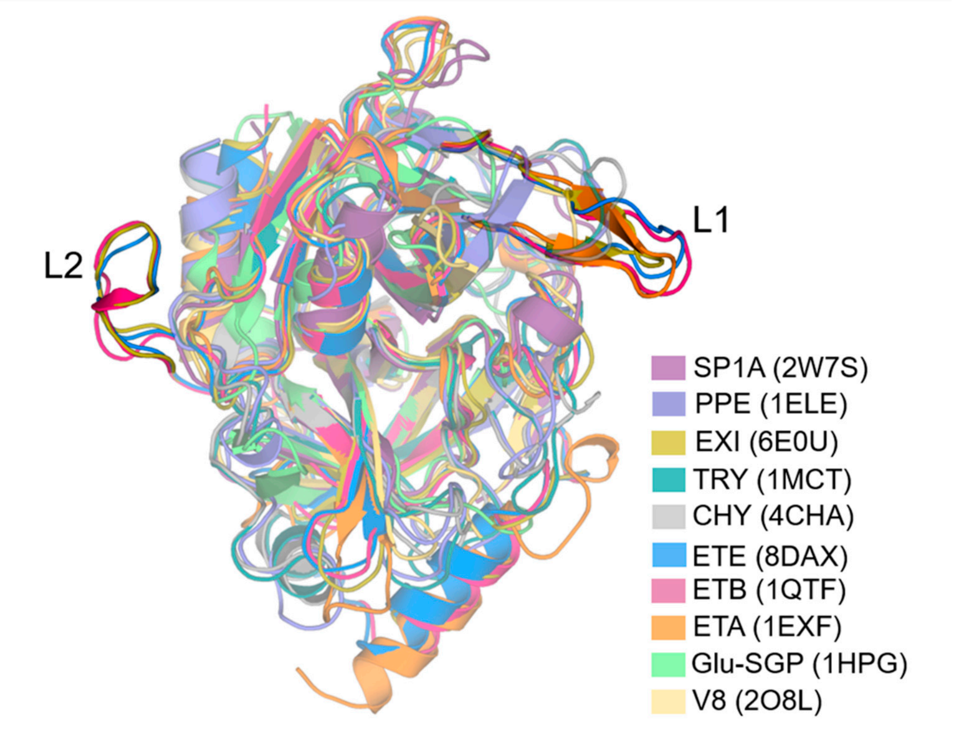

Figure S10. Structural alignment of different serine proteases. Loops L1 and L2 are highlighted. TRY, CHY and PPE stand for trypsin, chymotrypsin and porcine pancreatic elastase, respectively. The PDB ID of each structure is shown in parentheses. Proteins were structurally aligned at https://modbase.compbio.ucsf.edu/salign/.

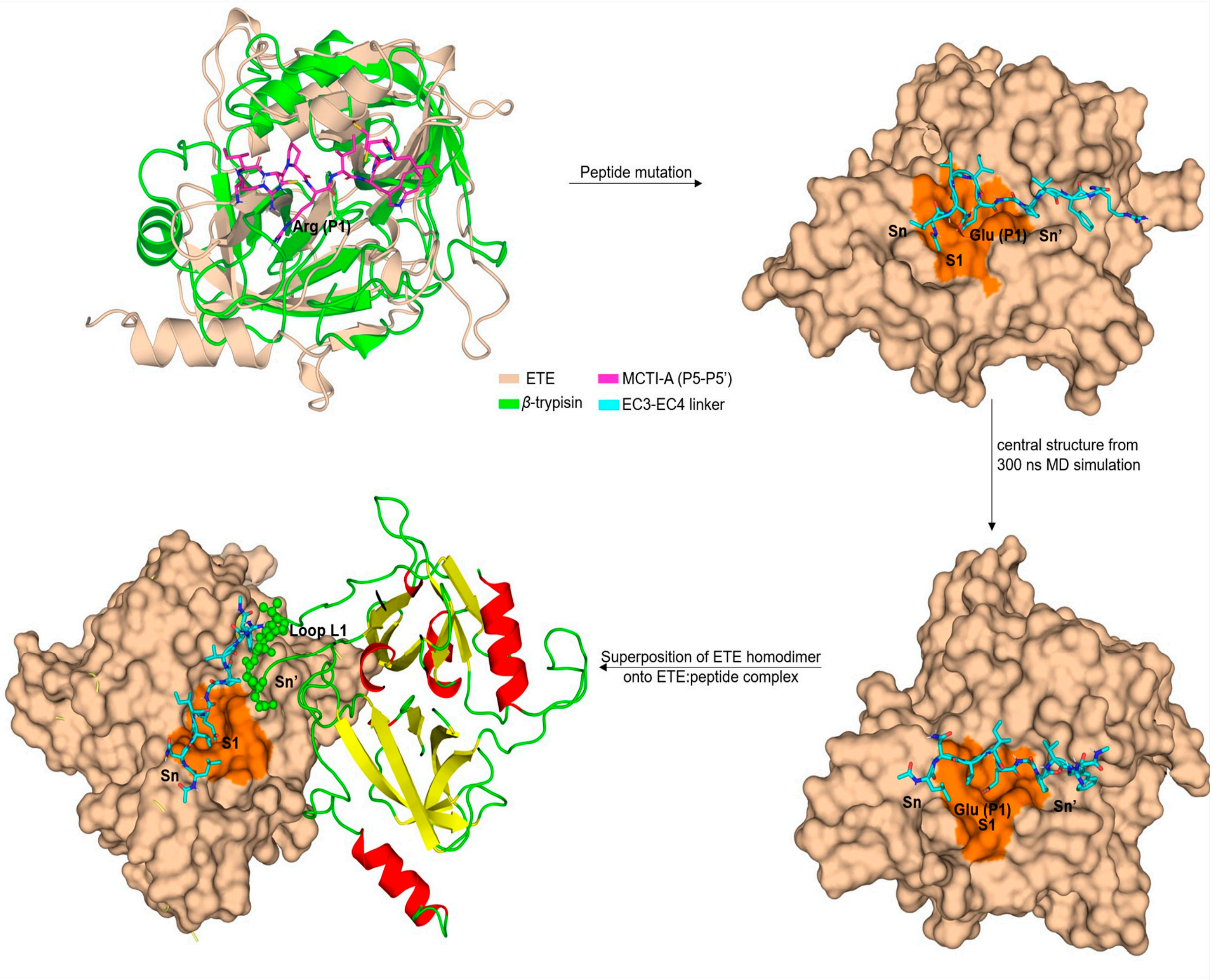

Figure S11. Model of ETE in complex with Dsg1 EC3-EC4 linker and superposition with the predicted structure of ETE homodimer. The crystal structures of ETE with an active oxyanion hole and porcine *β*-trypsin in complex with the peptide inhibitor MCTI-A (PDB 1MCT) were superimposed. The MCTI-A residues from P5 to P5' were then mutated to match the sequence of the EC3-EC4 linker (LNVIE↓GSVFR) with the mutagenesis plugin of Pymol 2.1.0 [32]. The central structure of the ETE:peptide complex from a 300 ns MD simulation was obtained and the ETE homodimer (ETE_2-CP-0) was superimposed onto the former structure. Notice that loop L1 (residues shown in spheres) inserts into the Sn' side of ETE active site, which sterically hinders the linker accommodation at the Sn' side of the active site when ETE is in the dimeric form. Due to the C2 symmetry of the ETE_2-CP-0 structure, the Sn' side of the second active site (not represented for brevity's sake) is also occupied by loop L1.

Text S2. Preparation of *N*-Tertbutyioxycarbonyl glutamic acid *O*-phenyl ester (Boc-L-Glu-OPh)

*Synthesis ester **2**.* A solution of Boc-L-Glu-(OBn)-OH **1** (2.01 g, 5.95 mmol), phenol (557 mg, 5.92 mmol), dicyclohexylcarbodiimide (DCC) (1.219 g, 5.91mmol) and pyridine (491 mg, 6.2 mmol) in 30ml of ethyl acetate, was stirred at room temperature for 14 hours (step 1, Figure S12). The precipitated dicyclohexylurea was filtered. The filtrate was washed with saturated $NaHCO_3$ solution, 10% citric acid solution and water respectively. The organic layer dried over Na_2SO_4, filtered, and the solvent removed under reduced pressure. The crude mixture was submitted to flash chromatography (SiO_2, 5:1 hexane-ethyl acetate) gave (750 mg, 1.81 mmol) (30%) of **2**. Spectral data were identical to the reported data [28].

^{1}H NMR (400MHz) ($CDCl_3$) : 1.45 (s, 9H), 2.09-2.18 (m, 1H), 2.38-2.42 (m, 1H), 2.50-2.64 (m, 2H), 4.57 (m, 1H), 5.14 (s, 2H), 5.13-5.20 (m, 1H), 7.08-7.34 (m, 10H)

*Synthesis of Boc-L-Glu-OPh (**3**).* The product **2** was dissolved in 12 ml of methanol, followed by the addition of 75 mg of Pd/C (10% w/w). The flask was closed with a rubber septum and a balloon with hydrogen was attached. This mixture was stirred at room temperature for 3 hours (step 2, Figure S12). After this time, the catalyst was removed by filtration under CELITE and the solvent evaporated under reduced pressure gave (286 mg, 0.9 mmol) (50%) of **3**. Spectral data were identical to the reported data [72].

^{1}H NMR (250MHz) ($CDCl_3$) MHz ^{1}H NMR ($CDCl_3$) 1.46 (s, 9H), 2.13-2.16 (m, 1H), 2.36 (s, 1H), 2.5-2.57 (m, 2H), 4.57 (d, 1H), 5.22-5.25 (m, 1H), 7.08-7.38 (m, 5H).

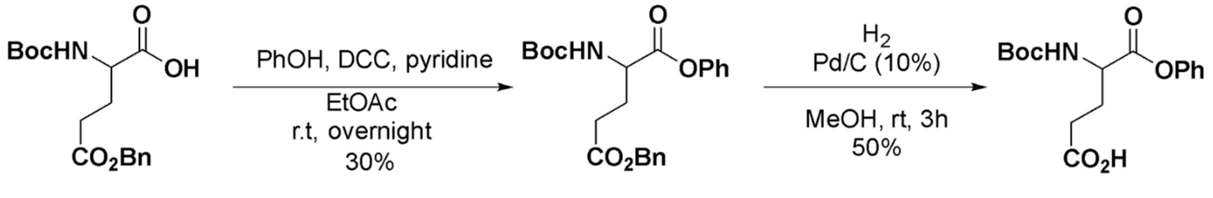

(1) (2) (3)

Figure S12. Synthesis of Boc-L-Glu-OPh. Steps are described in Text S2.

Text S3. Details of the Umbrella sampling free energy calculation protocols

The last frames of the 100 ns trajectories of the complexes with ΔG_{eff} < -31 kcal/mol and the central structures obtained from 1 μs MD simulations were chosen to conduct US free energy calculations (Figure 2) in order to predict the lowest-energy pose [65, 73]. The proteins were pulled along the *z* axis after careful manual orientation to prevent clashes. In total, 23 windows separated by 1 Å were created by displacing the second protein chain using the program *gmx editconf* of Gromacs 5.1.4 [74]. Two of the previous windows were obtained by approaching both proteins 2 and 1 Å, respectively, relative to the equilibrium position, i.e., that of the initial structure, while the remaining windows spanned distances between the reference atoms (see below) ranging from 0 to 20 Å along *z.* The six external degrees of freedom (DOFs) of each protein chain, i.e., the rotational and translational motions, were prevented by attaching the proteins to three dummy lead-like particles (D1, D2 and D3) that were kept fixed in the simulation box by means of tight position restraints (k= 100 kcal·mol^{-1}·Å^{-2}) [66, 75]. The attachment of each chain to the fixed dummy particles involved a distance restraint (D1-G192(Cα), k= 10 kcal·mol^{-1}·Å^{-2}), two angular restraints (D2-D1-G192(Cα) and D1- G192(Cα)-I151(Cα), k= 200 kcal·mol^{-1}·rad^{-2}) and three dihedral restraints (D3-D2-D1- G192(Cα), D2-D1-G192(Cα)-I151(Cα) and D1- G191(Cα)-I151(Cα)-S168(Cα), k= 200 kcal·mol^{-1}·rad^{-2}), with the restraint equilibrium values being set to those initially calculated for every window of the different systems. Please, note that the previous k values must be halved when set in the restraint inputs of Amber 20 [58].

Each window was solvated in a cuboid box with edges along the *z* direction being placed, at least, 12 Å away from the protein surface to prevent interaction with copies in neighboring cells as the partners were separated within the simulation box. In the orthogonal axes, the edges were placed 8 Å away from the proteins. HMR was employed to increase the integration timestep from 2 to 4 fs during the production runs [62]. All windows were simulated in duplicate for 50 ns after appropriate EM and equilibration steps, which were identical to those reported for the conventional MD simulations, except for the gradual release of the restraints on the protein heavy atoms, which was not conducted in this case.

The distance profile between D1 and G192(Cα) of the second chain, i.e., the one pulled along the *z* axis, was collected for every window and potentials of mean force (PMFs) were obtained from these distance distributions using weighted histogram analysis (WHAM) with the wham program available at Grossfield lab's website (http://membrane.urmc.rochester.edu/?page_id=126). The first 10 ns of each production run were discarded prior to these calculations.

US free energy calculations were performed to predict the PMFs associated with the rotation around the N-Cα-C-O dihedral (ξ) of residue P186 of ETE. The N-Cα-C-O dihedral was manually rotated from -180 to 175° in 5° strides, totalizing 72 windows, and was restrained to each equilibrium value by means of a dihedral restraint with k=200 kcal·mol^{-1}·rad^{-2}. As before, EM and equilibration steps were carried out for each window, followed by a 50 ns production run. Hydrogen mass repartitioning was used to increase the performance. PMFs were calculated as explained in the previous paragraph, but this time on the basis of the collected N-Cα-C-O dihedral distributions for every window. All MD simulations were conducted five times and SEMs associated with each PMF point are reported as error bars in the graphs.
